# High quality genome of *Erigeron breviscapus* provides a reference for herbal plants in Asteraceae

**DOI:** 10.1111/1755-0998.13257

**Published:** 2020-10-22

**Authors:** Simei He, Xiao Dong, Guanghui Zhang, Wei Fan, Shengchang Duan, Hong Shi, Dawei Li, Rui Li, Geng Chen, Guangqiang Long, Yan Zhao, Mo Chen, Mi Yan, Jianli Yang, Yingchun Lu, Yanli Zhou, Wei Chen, Yang Dong, Shengchao Yang

**Affiliations:** ^1^ State Key Laboratory of Conservation and Utilization of Bio‐Resources in Yunnan The Key Laboratory of Medicinal Plant Biology of Yunnan Province National and Local Joint Engineering Research Center on Germplasm Innovation and Utilization of Chinese Medicinal Materials in Southwest China Yunnan Agricultural University Kunming China; ^2^ Province Key Laboratory Biological Big Data College Yunnan Agricultural University Kunming China; ^3^ Institute of Primate Translational Medicine Kunming University of Science and Technology Kunming China; ^4^ State Key Laboratory of Plant Physiology and Biochemistry College of Life Sciences Zhejiang University Hangzhou China; ^5^ Plant Germplasm and Genomics Center The Germplasm Bank of Wild Species Kunming Institute of Botany Chinese Academy of Sciences Kunming China

**Keywords:** *Erigeron breviscapus*, genome, GWAS, scutellarin

## Abstract

*Erigeron breviscapus* is an important medicinal plant in Compositae and the first species to realize the whole process from the decoding of the draft genome sequence to scutellarin biosynthesis in yeast. However, the previous low‐quality genome assembly has hindered the optimization of candidate genes involved in scutellarin synthesis and the development of molecular‐assisted breeding based on the genome. Here, the *E. breviscapus* genome was updated using PacBio RSII sequencing data and Hi‐C data, and increased in size from 1.2 Gb to 1.43 Gb, with a scaffold N50 of 156.82 Mb and contig N50 of 140.95 kb, and a total of 43,514 protein‐coding genes were obtained and oriented onto nine pseudo‐chromosomes, thus becoming the third plant species assembled to chromosome level after sunflower and lettuce in Compositae. Fourteen genes with evidence for positive selection were identified and found to be related to leaf morphology, flowering and secondary metabolism. The number of genes in some gene families involved in flavonoid biosynthesis in *E. breviscapus* have been significantly expanded. In particular, additional candidate genes involved in scutellarin biosynthesis, such as flavonoid‐7‐*O*‐glucuronosyltransferase genes (*F7GATs*) were identified using updated genome. In addition, three candidate genes encoding indole‐3‐pyruvate monooxygenase YUCCA2 (YUC2), serine carboxypeptidase‐like 18 (SCPL18), and F‐box protein (FBP), respectively, were identified to be probably related to leaf development and flowering by resequencing 99 individuals. These results provided a substantial genetic basis for improving agronomic and quality traits of *E. breviscapus*, and provided a platform for improving other draft genome assemblies to chromosome‐level.

## 1 INTRODUCTION

The Compositae is a large plant family containing 25,000–30,000 species that accounts for approximately 10% of all angiosperms (Reyes‐Chin‐Wo et al., 2017). Many species in Compositae have considerable medicinal, edible, ornamental, and economic importance (Vidic et al., 2016), such as *Centaurea cyanus* and *Artemisia annua*, which were the first large medicinal plants used in Europe and America, respectively. *Erigeron breviscapus* is a medicinal plant with great potential in Compositae. Scutellarin, a major active component of flavonoids abundant in its leaves, is widely used as a prescription drug in treating cardiovascular and cerebrovascular diseases (PPRC, 2015). Breviscapine injection (>90% scutellarin) and *E. breviscapus* injection (about 16.7% scutellarin) currently prepared from *E. breviscapus* extracts (PPRC, 2015; Renwei et al., 2011), with a total annual output value over 5 billion yuan, have considerable economic benefits and application value. Based on the previous draft genome assembly and engineering yeast for the production of breviscapine (scutellarin and apigenin‐7‐*O*‐glucuronide) by genomic analysis and synthetic biology (Liu et al., 2018; Yang et al., 2017), *E. breviscapus* has becoming the first medicinal plant accomplishing the process from genome to biosynthesis. However, due to the high heterozygosity and repeatability in the *E. breviscapus* genome, Illumina short‐read sequencing used in the previous version is not sufficient for high quality genome assembly, thus hindering the complete capture of candidate genes involved in scutellarin synthesis and development of molecular‐assisted breeding based on the genome.

To date, genomes from many species in Asteraceae have been released, including horseweed (*Conyza canadensis*), globe artichoke *(Cynara cardunculus* var. *scolymus),* safflower (*Carthamus tinctorius* L.), fleabanes (*E. breviscapus*), lettuce (*Lactuca sativa*), sunflower (*Helianthus annuus* L.), sweet wormwood (*A. annua*), chrysanthemum (*C. nankingense* and *C. seticuspe*), cup plant (*Silphium perfoliatum*) and milk thistle (*Silybum marianum*; Peng et al., 2014; Scaglione et al., 2016; Bowers et al., 2016; Yang et al., 2017; Reyes‐Chin‐Wo et al., 2017; Badouin et al., 2017; Shen et al., 2018; Song, et al., 2018; Hirakawa et al., 2019; https://www.ncbi.nlm.nih.gov/genome/?term=Asteraceae). Among them, only sunflower and lettuce in Compositae have reached the chromosome level (Badouin et al., 2017; Reyes‐Chin‐Wo et al., 2017). PacBio RSII sequencing combined with Hi‐C technology provides the ability to generate long reads that effectively bridge complex regions, such as repeat sequences, and generate long‐range haplotypes to distinguish genes within gene families, thus improving the quality of assembly and helping to anchor a large number of genomic fragments at the chromosome level. Therefore, it is necessary to update the genome of *E. breviscapus* using third‐generation sequencing and Hi‐C technology.

Flavonoids are a large class of plant secondary metabolites (Jaakola & Hohtola, 2010). Accumulation of flavonoids is a strategy to cope with adverse environmental stresses in adapting to special environments, with flavonoids functioning to protect plants from oxidation, ultraviolet radiation and pathogen invasion (Petrussa et al., 2013; Singh et al., 2017; Wang et al., 2011). *E. breviscapus* is widely distributed in middle and high‐altitudes, and shows high accumulation of scutellarin, as well as many flavonoids including luteolin, kaempferol, quercetin and hesperetin in leaves (Chu et al., 2005; Su et al., 2001). As far as the plant itself is concerned, it will be meaningful to explore whether this accumulation is related to changeable climate factors in middle and high‐altitude habitats, such as low oxygen, ultraviolet radiation, and temperature differences between day and night, and is also reflected in the evolution of *E. breviscapus* genome. In addition, the leaf number is the basis for ensuring high yield during the planting of *E. breviscapus* (Song et al., 2018). The lack of knowledge on mechanisms regulating flowering, self‐incompatibility and leaf number in *E. breviscapus* restricts breeding late flowering and leafy varieties (Zhang et al., 2015). Therefore, resequencing based on a high quality reference genome is a potential and effective method to develop markers for molecular assisted breeding (Cao et al., 2016; Huang et al., 2010).

In this study, we used the third generation of genome sequencing technology to update the genome assembly of *E. breviscapus*. On the basis of the obtained high‐quality assembled genome, we resequenced 99 individuals to identify single nucleotide polymorphisms (SNPs) and candidate genes controlling late flowering and number of leaves, re‐captured candidate genes involved in major flavonoids biosynthesis, and established a stable genetic transformation system. The results will provid valuable technical support and genetic resources for genetic improvement and optimization of scutellarin biosynthesis in *E. breviscapus* in the future, and also provide a template for improvement of other draft genomes to chromosome‐level.

## 2 MATERIALS AND METHODS

### 2.1 Genome sequencing and assembly

The same seedling used for *E. breviscapus* genomic sequencing was provided by Longjin Pharmaceutical Co., Ltd, and planted in the greenhouse at Yunnan Agricultural University. Total genomic DNA was extracted from fresh leaves of 60‐day‐old plants using the GenElute Plant Genomic DNA Miniprep Kit (Sigma‐Aldrich). 40 μg of sheared DNA was used to construct SMRT Cell libraries with an insert size of 17 kb. These libraries were sequenced in SMRT DNA sequencing cells with P6/C4 chemistry. Together with the long reads obtained from the previous study (Yang et al., 2017), about 157.41 Gb of raw data was obtained on a PacBio RSII instrument. Illumina paired‐end sequencing of 100 bp with insert sizes ranging from 150 to 800 bp and mate pair sequencing with insert sizes from 2 to 20 kb were carried out as previously reported (Yang et al., 2017).

After filtered by minimum length of 50 bp and trimmed of adapter sequence by home‐made scripts, de novo assembly of the PacBio reads was performed with the default wtdbg pipeline (https://github.com/ruanjue/wtdbg). Illumina reads were trimmed by software trimmomatic‐0.36 with default parameters and were used to correct base‐calling by proovread with the default parameters (Hackl et al., 2014).

### 2.2 Hi‐C assisted contig clustering

The Hi‐C library was prepared with the standard procedure of Novogene as follows. Nuclear DNA was cross‐linked in situ, extracted and then digested with a restriction enzyme. The sticky ends of the digested fragments were biotinylated, diluted and then ligated to each other randomly. After being enriched and sheared again, biotinylated DNA fragments ranging from 400 to 500 bp were PE‐100 sequenced using Illumina HiSeq platform, producing 106.32 Gb of raw data. Raw Hi‐C reads were first trimmed by software trimmomatic‐0.36 with parameters “PE ‐threads 40 ‐phred33 TAILCROP: 70 MINLEN: 45 LEADING: 3 TRAILING: 3 SLIDINGWINDOW: 4:15”, the cleaned data were then aligned to the assembled contigs by bowtie2 (2.2.6) integrated in HiC‐Pro (2.9.0; Servant et al., 2015). Contigs were clustered onto chromosomes with LACHESIS with the following parameters ‘RE_SITE_SEQ = GATC, USE_REFERENCE = 0, OVERWRITE_GLM = 1, OVERWRITE_CLMS = 1, CLUSTER_N = 9, CLUSTER_MAX_LINK_DENSITY = 3’ (Burton et al., 2013). The completeness of the final assembly was assessed by the analysis of Benchmarking Universal Single‐Copy Orthologues (BUSCO; Waterhouse et al., 2017) with Embryophyta odb9 database and default parameters.

### 2.3 Transcriptome sequencing

To investigate the expression patterns of candidate genes involved in flavones and caffeoylquinic acids biosynthesis, the tissues collected from the roots, stems, leaves and flowers of 90‐day‐old plants were used for tissue specificity analysis. Each plant represents a biological repeat, and three biological replicates were performed. To examine the effect of abscisic acid (ABA), salicylic acid (SA) and gibberellin (GA) hormones on gene expression, 60‐day‐old plants were treated with 200 μmol/l ABA, SA and GA by foliar spray, and then leaves harvested at 4, 12, and 24 hr after treatment, respectively. Five to eight leaves from different plants were mixed and represent a biological repeat, and three biological replicates were performed. All collected samples were immediately frozen in liquid nitrogen and stored at −80°C until use. Total RNA was extracted using Qiagen RNeasy Plant Mini Kits. According to the manufacturer's instructions, total RNA‐seq libraries were prepared using TruSeq RNA Library Preparation Kit, v. 2 (Illumina), and were subsequently pair‐end sequenced with a read length of 150 bp on the HiSeq 4000 platform. In total, about 688.9 billion RNA‐seq reads were obtained, representing about 103.3 Gb of raw data. According to the following metrics: (a) to remove adaptors; (b) to trim 3′ end bases with a quality score below 20; and (c) to remove low‐quality reads with base Ns or more than 10% of bases with a quality score below 20, raw RNA‐seq reads were trimmed and filtered by Trimmomatic (Bolger et al., 2014). The clean RNA‐seq reads were aligned to the *E. breviscapus* genome assembly using TopHat (v. 2.0.10; Trapnell et al., 2012) with default parameters and the fragments per kilobase of transcript per million fragments mapped (FPKM) value was calculated for each protein‐coding gene by Cufflinks (v. 2.1.1; Trapnell et al., 2012) using default parameters. FPKM > 0.05 was used as the cutoff value to identify expressed genes.

### 2.4 Estimation of the genome heterozygosity and repeat content by *k‐mer* analysis

The quality‐filtered short fragments from the Illumina platform were subjected to *17‐mer* frequency distribution analysis with Jellyfish (v.2.2.5; Marçais & Kingsford, 2011) and Genomic Character Estimator program (GCE v1.0.0; https://github.com/nottwy/genome‐character‐estimator).

### 2.5 Annotation and gene‐model prediction

For repeat annotation, Tandem Repeat Finder (v. 4.07b; Benson, 1999) was used to identify tandem repeats in the genome assembly. RepeatMasker (v. 4.0.5) and RepeatProteinMasker (Tarailo‐Graovac & Chen, 2009) were applied to search for transposable elements against Repbase library (v. 18.07; Jurka et al., 2005). These results were then combined with the de novo prediction using LTR_FINDER (v.1.05; Xu & Wang, 2007) and RepeatModeler (v. 1.0.8; Tarailo‐Graovac & Chen, 2009).

The software tRNAscan‐SE (v. 1.3.1; Lowe & Eddy, 1997) with default parameters for eukaryotes was used for tRNA annotation. Homology‐based rRNA annotation was performed by mapping plant rRNAs (downloaded from Rfam database; Burge et al., 2012) to the *E. breviscapus* genome using BLASTN with parameters of “E‐value = 1e^−5^”. miRNA and snRNA genes were predicted by INFERNAL (v. 1.1; Nawrocki et al., 2009) using the Rfam database (release 11.0; Gardner et al., 2010).

In the present study, we combined three different gene‐model prediction methods, homology‐based predictions, de novo predictions, and transcriptome‐based predictions. In the homologue‐based gene‐prediction model, protein sequences of *A. annua*, *Cynara cardunculus*, *H. annuus*, *L. sativa*, *Arabidopsis thaliana*, *Nicotiana tabacum*, *Oryza sativa* and *Vitis vinifera* downloaded from the National Centre for Biotechnology Information (https://www.ncbi.nlm.nih.gov/) and were subjected to TBLASTN analysis to the *E. breviscapu* assembled genome with a cutoff E‐value of 1e^−5^ (Altschul et al., 1990). BLAST hits corresponding to reference proteins were concatenated by Solar after low‐quality records were removed. The genomic sequence of each reference protein was extended upstream and downstream by 2,000 bp to represent a protein‐coding region. For de novo prediction, we used AUGUSTUS (v. 2.5.5; Stanke et al., 2006), GENSCAN (v. 1.0; Cai et al., 2014), SNAP (released 29 November 2013; Korf, 2004), and glimmerHMM (v. 3.0.2) on the repeat‐masked genome, with parameters trained from *A. thaliana*. In the transcriptome‐based prediction, unigenes identified by TopHat (v. 2.0.10; Trapnell et al., 2012) were first aligned to the genome assembly and were then integrated with PASA (Haas et al., 2003) with default parameters. All predicted gene structures were integrated into a consensus set with EVidenceModeler (EVM; Haas et al., 2008). Genes were then annotated according to homologous alignments with BLAST (E*‐*value ≤ 1e^−5^) against several databases including the nr (Marchler‐Bauer et al., 2010) databases of NCBI, Swiss‐Prot, and TrEMBL. We further used InterProScan (v4.3; Hunter et al., 2011) to predict domain information and gene ontologies (GO terms; Dimmer et al., 2011). KAAS (Kanehisa & Goto, 2000) was used for KEGG pathway annotation.

### 2.6 Gene families analysis

To construct the phylogenetic tree and conduct the divergence time estimation and gene expansion/contraction analysis, OrthoMCL (v. 2.0.9; Li et al., 2003) pipeline with the settings (BLASTP E‐value < 1e^−5^) was applied to identify the potential orthologous gene families among *E. breviscapus*, *A. annua*, *C. cardunculus*, *H. annuus*, *L. sativa*, *A. thaliana*, *N. tabacum*, *O. sativa* and *V. vinifera*. To construct the phylogenetic tree, MUSCLE (v.3.8.31; Edgar, 2004) with default settings was used to align single‐copy orthologous gene sequences from nine species, and PhyML (v. 3.0) with default parameters was subsequently used to construct the tree. In addition, the known divergence time from the public resource TIMETREE (http://www.timetree.org) was provided for calibration and the program MCMCtree from the PAML package (Yang, 2007) was applied to estimate the divergence time. Based on the phylogeny and gene family size, CAFE (v.2.1; De Bie et al., 2006) was applied to identify gene families which had undergone expansion and/or contraction with the parameters “*p* = .05, number of threads = 10, number of random = 1,000, and search for lambda”.

To detect genes under positive selection, we used the coding DNA sequence (CDS) libraries of *H. annuus* and *A. annua* to run BLASTN against the *E. breviscapus* CDS library, respectively. The best hits were analysed in *KaKs*_Calculator v.2.0 (Zhang et al., 2006) with default parameters.

The specific gene families of seven species (*V. vinifera*, *L. sativa*, *H. annuus*, *E. breviscapus*, *A. annua*, *A. thaliana*, and *O. sativa*) were identified using the HMMER3 (http://hmmer.janelia.org/) software and the Pfam‐A data sets was downloaded from PFAM (http://pfam.janelia.org/). The seed file for CHI domain (PF02431) was obtained, and Pfam‐A data sets for six other types, namely *PAL*, *CHS*, *4Cl*, *F6H*, *FSII*, and *F7GAT* were transferred from fasta sequences downloaded from NCBI to stockholm file by perl script “fasta2sto.pl”. The domain file was used as the first template to scan the gene family whereby the output genes were filtered out with an E‐value below 1e‐10. The filtered genes were used as second templates for a second round of scanning of the target gene families. Similarly, the second phase of output genes was filtered out with an E‐value of 1e‐10. The putative genes from the gene family were identified. Finally, the gene families of all seven species were filtered by blast with the downloaded sequence with cutoff: identity ≥ 70%.

### 2.7 Sampling and resequencing

A total of 99 *E. breviscapus* samples, including 49 with low leaf number (≤20) and 50 with high leaf number (>120), were selected from the greenhouse of Longjin Pharmaceutical Co., Ltd. For phenotypic evaluation, three traits including branch number, leaf number and plant weight were counted. Young leaves collected from single individuals were immediately frozen in liquid nitrogen. Total DNA was extracted with the DNAsecure plant kit and at least 2 µg of genomic DNA for each sample was used to construct a sequencing library. 72‐ single‐end 100bp (from 50 high‐leaf and 22 low‐leaf individuals) and 27 paired‐end 150 bp (all from low‐leaf individuals) sequencing libraries with an insert size of approximately 400 bp were sequenced on BGI‐500 and Illumina HiSeq 4000 sequencer, respectively (Table S16). Raw reads were filtered using NGSQCToolkit_v2.3.3 (Patel & Jain, 2012), where reads containing adapter or poly‐N, and low‐quality reads (reads with >30% bases having Phred quality ≤25) were removed.

### 2.8 Sequence alignment, variation calling and annotation

All the clean reads for each sample were mapped to the newly updated genome with Burrows–Wheeler Aligner program (BWA, Ver. 0.7.10‐r789; Li & Durbin, 2009) with default parameters. We sorted alignments according to the mapping coordinates, converted mapping results into the BAM format and filtered the unmapped and nonunique reads with SAMtools (Ver. 1.3.1; Li et al., 2009) software. Duplicated reads were filtered with the Picard package (picard.sourceforge.net, Version: 2.1.1). Reads around Indels were realigned with Genome Analysis Toolkit (GATK, Ver. 3.3‐0‐g37228af; McKenna et al., 2010) in the following two steps: package RealignerTargetCreator was firstly used to identify regions where realignment was needed; package IndelRealigner was then used to realign the regions found in the first step, which produced a realigned BAM file for each accession.

The variation detection followed the best practice workflow recommended by GATK. In brief, variants were called for each sample by the GATK HaplotypeCaller (Emanuelli et al., 2013). A joint genotyping step for comprehensive variations union was performed on the GVCF files. Raw SNPs were filtered by VCFtools (v0.1.13; Danecek et al., 2011) with the following parameters “QD < 2.0 || FS > 60.0 || MQ < 40.0 || MQRankSum < −12.5 || ReadPosRankSum < −8.0”. Indels that shorter than or equal to 10 bp were filtered with “QD < 2.0 || FS > 200.0 || ReadPosRankSum < −20.0”. SNPs and Indels that were not biallelic, had >5% missing calls and MAF < 0.05 were removed. The identified SNPs and Indels were further annotated with ANNOVAR software (Wang et al., 2010) and divided into groupings of variations occurring in intergenic regions, coding sequences and introns, on the basis of newly updated *E. breviscapus* genome annotation information.

### 2.9 Population genetic analysis

The whole‐genome SNPs were used to construct the ML (Maximum likelihood method) phylogenetic tree with 100 bootstrap using SNPhylo (Ver. 20140701; Clark et al., 2007). The tool iTOL (Letunic & Bork, 2019; http://itol.embl.de) was used to colour the phylogenetic tree. Principal component analysis (PCA) was performed with the Genome‐wide Complex Trait Analysis (GCTA, Ver. 1.25.3) software (Yang et al., 2011), and the first three eigenvectors were plotted.

### 2.10 Genome‐wide association study analysis

A total of 4,255,459 high‐quality SNPs and 1,646,738 Indels identified in 99 *E. breviscapus* samples were used to perform SNP‐level and Indel‐level genome‐wide association study (GWAS) for three traits, respectively. GWAS was performed with a linear mixed model (LMM) in genome‐wide efficient mixed model association (GEMMA, Version: 0.98.1) software (Zhou & Stephens, 2012), whereas the estimated standardized relatedness matrix (‐gk 2) estimated by GEMMA (Zhou & Stephens, 2012) was used as a random effect to correct the population structure. To control the genome‐wide type I error rate, the effective number (N) of independent SNPs and Indels were calculated using the Genetic Type I Error Calculator (GEC, v0.2; Browning & Browning, 2016). Significant (0.05/N, Bonferroni correction) and suggestive (1/N) *p*‐value thresholds were set as 2.09e‐8 and 4.19e‐7 for SNPs and 5.48e‐8 and 1.09e‐6 for Indels, respectively. The genomic inflation factor (lambda) was calculated by R package qqman (Turner, 2014). All software parameters used in our study are summarized in Table S19.

### 2.11 Annotation and analysis of UDP‐glycosyltransferase genes

The UDP‐glycosyltransferase (UDPGT) genes in the *E. breviscapus* genome were predicted by hmmsearch with the UDPGT hmm model PF00201 (E‐value < 1e^−10^) from Pfam (Finn et al., 2011; Finn et al., 2011) and the putative UDPGT proteins were screened by amino acid length between 400 and 650. A total of 144 UDPGT genes were predicted in the *E. breviscapus* genome. Based on the previous study, the maximum‐likelihood tree was constructed by MEGA (Kumar et al., 2016), 144 UDPGT genes were then clustered into several classes.

### 2.12 Overexpression vectors construction and transformation of *E. breviscapus*

The full‐length open reading frames (ORFs) of *PAL*, *C4H*, *4Cl*, *CHS*, *CHI*, and *FSⅡ* were cloned from leaves of *E. breviscapus*. The target fragments and pCambia1301‐35SN were cut by the restriction enzymes as shown in Table S14. The purified DNA fragments were inserted into the linearized pCambia1301‐35SN with the T4 DNA ligase (NEB, Kunming, China). The resulting constructs were introduced into *Agrobacterium tumefaciens* strain EHA105 by electroporation. *E. breviscapus* transgenic plants were generated as described previously (Zhang et al., 2007).

### 2.13 Gene expression and high‐performance liquid chromatography analyses

The relative transcript levels of six genes located upstream of breviscapine biosynthesis were measured by reverse transcription quantitative real‐time PCR (qRT‐PCR). Total RNA was extracted from leaves of transgenic *E. breviscapus* using the RNAprep pure Plant Kit (Tiangen) and reverse transcribed into cDNA using the PrimeScript RT Master Mix (TaKaRa). PCR amplification was performed in a Roche LightCycler 96 (Roche) using the SYBR Green qRT‐PCR Master Mix (TaKaRa) according to the manufacturer's instructions. The thermal profile for SYBR Green qRT‐PCR was 95°C for 2 min, followed by 40 cycles of 95°C for 20 s, 58°C for 20 s, and 72°C for 20 s. The actin gene was chosen as a reference gene to control for normalization. Samples for high‐performance liquid chromatography (HPLC) analysis were prepared as described previously (Liu et al., 2018). The scutellarin standard was obtained from Solarbio. The experiments were carried out with three biological replicates.

## 3 RESULTS

### 3.1 Assembly of reference genome of *E. breviscapus*

**FIGURE 1:**
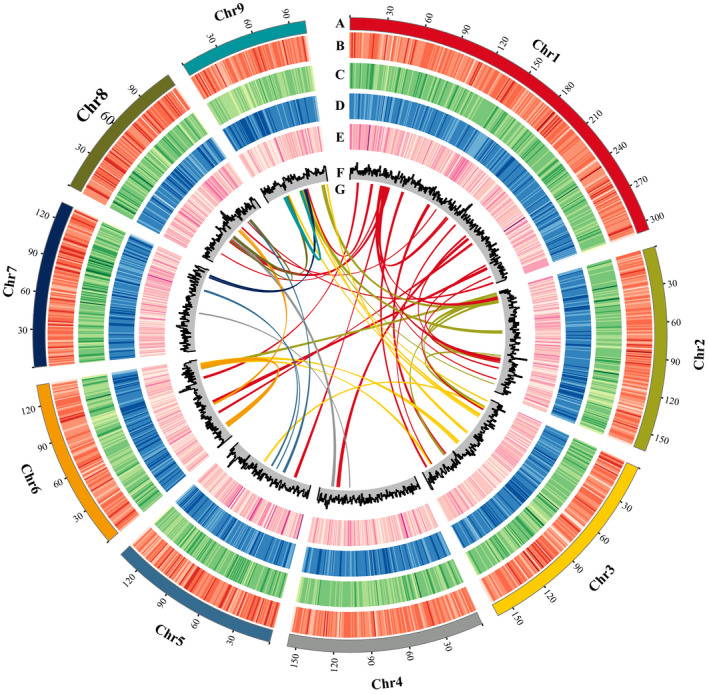
An overview of genomic features of *E. breviscapus*. (a) The genomic landscape of the nine *E. breviscapus* pseudo‐chromosomes. All density information was counted in nonoverlapping 1‐Mb windows; (b) SNP density; (c) Indel density; (d) repeat coverage; (e) gene density; (f) guanine‐cytosine (GC) content; (g) synteny relationship of gene blocks (with ≥10 homologous genes) between pseudo‐chromosomes [Colour figure can be viewed at wileyonlinelibrary.com]

According to the standard *17‐mer* curves, the heterozygosity of *E. breviscapus* was approximately 2.04% (Figure S1). In the previous study, a total of 320.50 Gb of Illumina paired‐end sequencing data (210.8X) were obtained (Yang et al., 2017). Here, 157.41 Gb of PacBio raw data (103.6X), and 106.32 Gb of Hi‐C data (69.9X) were generated to update this complex genome assembly (Table S1). The total genome assembly amounted to 1.41 Gb, consisting of 18,973 contigs with the longest length of 1,416,127 bp and contig N50 of 140,946 bp (Table S2). With the aid of Hi‐C sequence data, 99.2% of the sequences were anchored and oriented onto nine pseudo‐chromosomes, with a total size of 1.43 Gb, covering 94.1% of the genome size estimated by flow cytometry (Yang et al., 2017). The completeness of the genome assembly showed that 88.5% of the plant sets were identified as complete (1,274 out of the 1,440 BUSCOs; Table S3) (BUSCO; Simao et al., 2015). By integrating homology‐based and de novo approaches, 67.42% of the genome was predicted as transposable elements, among which long terminal repeats (LTRs) were the most abundant characterized elements, accounting for 40.15% of the genome, while 25.69% of that could not be classified into any known cluster (Tables S4 and S5). Next, combined with the transcriptome data from four tissues (leaf, flower, stem, and root) and three plant hormone treatments (ABA, GA, and SA), 43,514 protein‐coding gene models were obtained, with an average coding‐sequence length of 1.14 kb and an average of 5.3 exons per gene (Tables S6 and S7). Also identified were 906 miRNAs, 854 tRNAs, 266 rRNAs, and 818 snRNAs (Table S8). The previously published *E. breviscapus* genome was approximately 1.2 Gb, with contig and scaffold N50 sizes of 18.8 kb and 31.5 kb, respectively (Yang et al., 2017). By comparing assembly statistics for the genome of *E. breviscapus* with Asteraceae, the updated genome increased in size to 1.43 G, with a scaffold N50 of 156.82 Mb and contig N50 of 140.95 kb (Table 1). The contig N50 of updated *E. breviscapus* genome was shorter than that of sunflower, but longer than those of other Asteraceae genomes, including *L. sativa*, *A. annua*, *C. Nankingense*, *C. seticuspe* and so on (Table 1). The genomic characterization of the *E. breviscapus* genome including chromosome, GC content, gene number, repeat content, and SNP density is shown in Figure 1.

**Table 1:** Assembly statistics for the genome of *E. breviscapus* compared to Asteraceae

Species	*E. breviscapus*		*H. annuus*	*L. sativa*	*C. cardunculus*	*A. annua*
Genera	Erigeron		Helianthus	Lactuca	Cynara	Artemisia
Chromosome number (2n)	18		34	18	34	18
Assembly ID	V1	V2	HanXRQr1.0	Lsat_Salinas_v7	CcrdV1	ASM311234v1
Total sequence length	1,217,085,526	1,430,807,110	3,027,844,889	2,384,188,817	725,197,765	1,792,856,094
Number of scaffolds		1,812	1,528	11,474	13,588	39,400
Scaffold N50	31,461	156,822,787	178,899,001	1,769,135	125,941	104,891
Number of contigs	464,088	18,973	13,954	168,553	73,428	190,477
Contig N50	18,821	140,946	414,085	28,336	19,399	20,144

Species	*E. canadensis*	*C. tinctorius*	*C. nankingense*	*C. seticuspe*	*Silphium perfoliatum*	*Silybum marianum*
Genera	Conyza	Carthamus	Chrysanthemum	Chrysanthemum	Silphium	Silybum
Chromosome number (2n)	18	24	18	18		—
Assembly ID	ASM77593v1	Safflower1	—	CSE_r1.0	Sp_tGBS_contigs	ASM154182v1
Total sequence length	326,165,195	865,937,202	2,527,345,456	2,721,839,164	121.712 (Mb)	1,477.57 (Mb)
Number of scaffolds	—	2,195,958	—	354,212	—	—
Scaffold N50	—	1,976	—	44,741	—	—
Number of contigs	20,075	3,254,412	24,051	961,201	1,197,534	258,575
Contig N50	20,748	368	130,678	*7,793*	*125*	*6,967*

### 3.2 Evolution analysis

Both ortholog clustering and gene family clustering analyses were performed using OrthoMCL on all of the protein‐coding genes of *E. breviscapus*, *A. thaliana*, *O. sativa*, *N. tabacum*, *V. vinifera*, *H. annuus*, *L. sativa*, *C. cardunculus*, *A. annua*, *and G. max*. In *E. breviscapus*, 43,514 protein‐coding genes were comprised of 4,538 single‐copy orthologues, 9,372 multiple‐copy orthologues, 10,113 unique paralogues, 9,536 other paralogues, and 9,955 unclustered genes (Figure S2). A total of 33,559 protein‐coding genes can be clustered into 14,045 gene families, among which 2,024 were unique gene families (Table S9).

**FIGURE 2:**
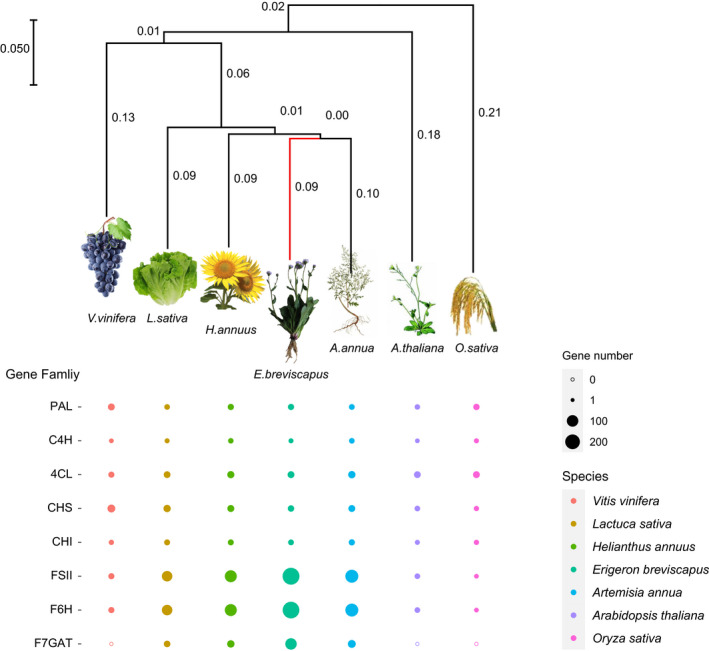
The phylogenetic relationships among seven plants and the number of genes involved in flavonoid biosynthesis. 4Cl, 4‐coumaroyl‐CoA ligase; C4H, cinnamate 4‐hydroxylase; CHI, chalcone isomerase; CHS, chalcone synthase; F6H, flavone‐6‐hydroxylase; F7GAT, flavonoid‐7‐O‐glucuronosyltransferase; FSI, flavone synthase I; FSII, flavone synthase II; PAL, phenylalanine ammonia lyase [Colour figure can be viewed at wileyonlinelibrary.com]

Based on a concatenated sequence alignment of single‐copy genes shared by the Asteraceae family and four other green plant species, a phylogenetic tree was constructed (Figure S3). As expected, *E. breviscapu* was clustered with other Asteraceae species such as *A. annua*, *H. annuus*, *L. sativa*, and *C. cardunculus* (Badouin et al., 2017; Reyes‐Chin‐Wo et al., 2017; Scaglione et al., 2016; Shen et al., 2018). The gene families that have expanded or contracted in the Asteraceae family were identified, and in total, 1986 gene families were expanded, whereas 6,764 gene families were contracted in *E. breviscapus* (Figure S3). The estimated divergence time point of *E. breviscapus* and *A. annua* was ~25.7 Ma. Further exploration of the number of genes in some gene families involved in flavonoid biosynthesis among other Compositae species and model plants found that the number of *C4H*, *4Cl*, *FSⅡ*,*F6H* and *F7GAT* genes in *E. breviscapus* have been significantly expanded, suggesting specific and diverse flavonoids synthesis in *E. breviscapus* (Figure 2; Table S10).

### 3.3 Positive selection of genes in *E. breviscapus*

An increased rate of nonsynonymous substitution (*Ka*) relative to synonymous substitution (*Ks*) within certain genes may account for the adaptive evolution of organisms at the molecular level (Qiu et al., 2012). Comparisons of orthologous gene pairs identified 344 genes in *E. breviscapus* versus *A. annua*, and 125 genes in *E. breviscapus* versus *H. annuus*, with a ratio of *Ka/Ks* significantly greater than 1.0 (*p*‐value < .05). A total of 14 genes were present in both lists of genes under positive selection (Table S11). These genes revealed candidates with putative functions related to leaf morphology, flowering and secondary metabolism. In particular, Cys(2) His(2) zinc finger transcription factors regulate leaf morphology (Chen et al., 2010) and MADS‐Box proteins control flowering in Arabidopsis (Favaro et al., 2003). Auxin‐responsive proteins regulate floral meristem maintenance and termination by repressing cytokinin biosynthesis and signaling (Zhang et al., 2018). The candidate genes of CYP450 and UGT family are involved in flavonoids biosynthesis (Liu et al., 2018; Noguchi et al., 2009).

### 3.4 Discovery of genes involved in flavones and caffeoylquinic acids biosynthesis

**FIGURE 3:**
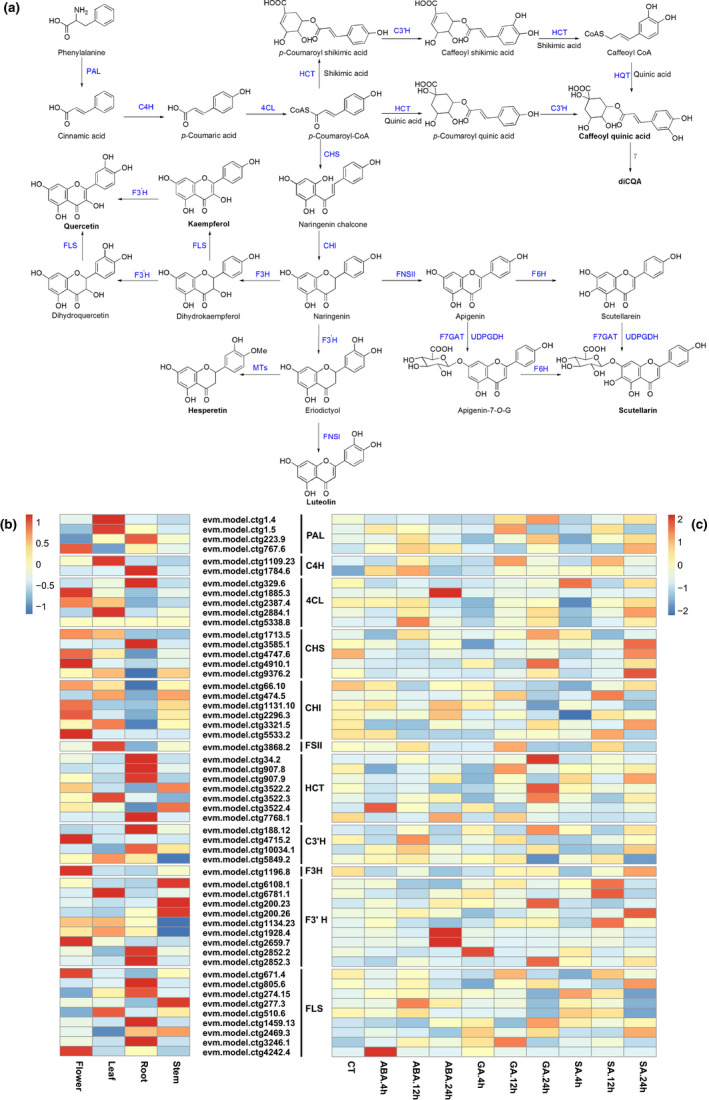
Discovery and expression analysis of genes involved in flavonoids and caffeoylquinic acids biosynthesis in *E. breviscapus*. (a) Proposed pathways for flavonoids and caffeoylquinic acids biosynthesis in *E. breviscapus*. Heatmaps show the expression patterns of candidate biosynthesis pathway gene in four tissues (b) and phytohormone‐treated leaf (c) from *E. breviscapus*. The information of used genes are provided in Table S12. The colour scale measures the FPKM value (fragments per kilobase of transcript per million fragments mapped) [Colour figure can be viewed at wileyonlinelibrary.com]

The major active component of *E. breviscapus* is breviscapine, mainly scutellarin, along with a small amount of apigenin 7‐*O*‐glucuronide. Flavonoid biosynthesis starts with phenylalanine, following catalysis by phenylalanine ammonia‐lyase (PAL), cinnamate‐4‐hydroxylase (C4H), 4‐coumaroyl‐CoA‐ligase (4Cl), chalcone synthase (CHS), chalcone isomerase (CHI), and flavone synthase II (FS II) to form apigenin, which is the precursor for breviscapine biosynthesis (Pandey et al., 2016). Caffeoylquinic acids (CQAs) are other active components of *E. breviscapus*, which have synergistic effect with scutellarin. CQA biosynthesis is also derived from the phenylpropanoid pathway as flavonoids, both of which share the same precursor *p*‐coumaroyl‐CoA, following catalysis by the BAHD acyltransferases family, which include hydroxycinnamoyl‐CoA:quinate hydroxycinnamoyl transferase (HQT) and hydroxycinnamoyl‐CoA:shikimate/quinate hydroxycinnamoyl transferase (HCT) (Legrand et al., 2016; Moglia et al., 2014, 2016) (Figure 3a; Table S12). Also *E. breviscapus* contains a few other flavonoids such as luteolin, kaempferol, quercetin, and hesperetin in *E. breviscapus*. Their biosynthetic pathways are shown in Figure 3a (Chen et al., 2018; Sun et al., 2018; Yuan et al., 2014; Table S12).

To investigate the expression patterns of candidate genes involved in the above biosynthetic pathways, we analysed the RNA‐seq data sets from the four tissues and plant hormone‐treated leaves of *E. breviscapus*. Most genes involved in scutellarin biosynthesis showed the highest expression in the leaves and the lowest in the stems. However, key genes involved in CQA biosynthesis exhibited the highest expression levels in the roots. Those genes related to biosynthesis of luteolin, kaempferol, quercetin, and hesperetin were differentially expressed in the roots, stems, leaves, and flowers (Figure 3b). While treated with plant hormone treatments, the expression levels of most genes involved in flavonoid and CQA synthesis significantly increased compared to the control. By prolonging the treatment, the expression of some genes continued to increase, whereas the others initially increased and then decreased (Figure 3c).

Our previous research had successfully identified two key enzymes involved in breviscapine biosynthesis: 1. F7GAT, which converts apigenin into apigenin‐7‐*O*‐glucuronide; and 2. F6H, which functions together with F7GAT in produce scutellarin from apigenin and synthesizes breviscapine de novo using engineered yeast (Chen et al., 2015; Jiang et al., 2014; Liu et al., 2018). Surprisingly, it mainly produced apigenin 7‐*O*‐glucuronide instead of scutellarin. The reason may be that only one *EbF7GAT* belonging to the UGT88X subfamily was identified from the previously published genome, which converted scutellarein to scutellarin (Liu et al., 2018). *EbF7GAT* has no strict specificity and thus recognizes many other flavonoids as its substrates. Here, we reanalysed the UGT gene family from the updated genome. A total of 144 UDPGT genes were identified and assigned to 19 gene families (Figure S4). Among these, three genes (*evm.ugt.ctg7693.8*, *evm.ugt.ctg3868.3* and *evm.ugt.ctg1608.6*) belong to the UGT88 gene family (Hirotani et al., 2000; Nagashima et al., 2000; Noguchi et al., 2009; Ono et al., 2010) (Figure S4a; Table S13). We subdivided these genes with genes in the UGT88 gene family reported in other plants, finding that two genes (*evm.ugt.ctg7693.8* and *evm.ugt.ctg3868.3*) were clustered together into the UGT88X branch (*evm.ugt.ctg3868.3* was the same with previously published genome), while another gene (*evm.ugt.ctg3868.3*) belongs to the UGT88F subfamily (Figure S4b).

### 3.5 Establishment of genetic transformation of *E. breviscapus*

Both overexpression and CRISPR/Cas9 gene editing need a stable genetic transformation system. Therefore, we established an Agrobacterium‐mediated genetic transformation system which involves inoculation, cocultivation, selection, differentiation and regeneration. To test the reliability of the genetic transformation system, six enzyme genes, including *PAL, C4H, 4Cl, CHS, CHI, and FS II* that locate in upstream of breviscapine biosynthesis were cloned from the updated genome and ligated each into a pCAMBIA1301‐35SN vector (Table S14). Transgenic plants were generated by Agrobacterium (EHA105)‐mediated leaf disc transformation (Figure S5a,b). The expression level of *PAL, C4H, 4Cl, CHS, CHI* and *FS II* was dramatically increased in the transgenic *E. breviscapus* plants, reaching 4.0‐ to 12.8‐fold higher than wild‐type (Figure S5c). Meanwhile, scutellarin content significantly increased in all overexpressing transgenic lines, ranging from 0.23% to 0.41%, 1.92–3.42 times higher compared to that in wild‐type (0.12%; Figure S5d).

### 3.6 GWAS analysis based on high quality reference genome

**FIGURE 4:**
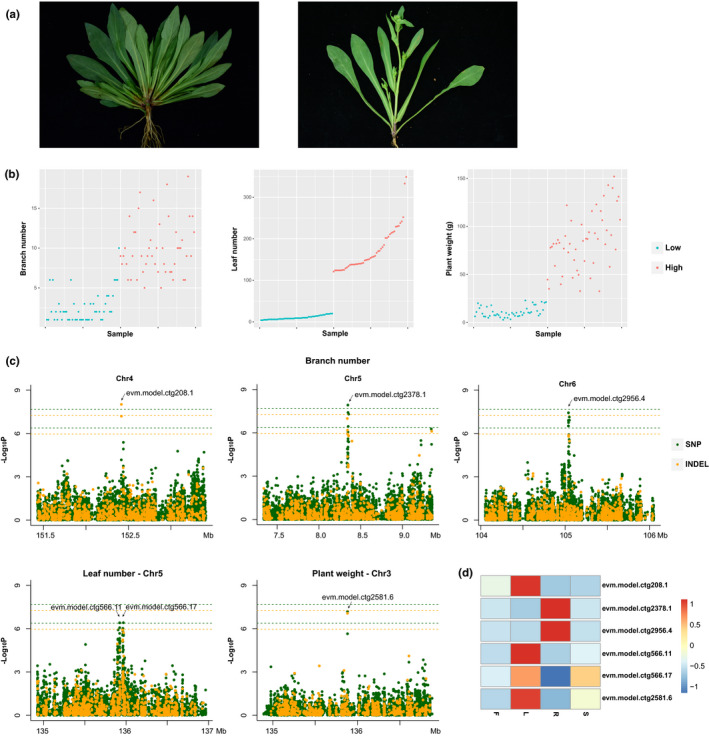
Phenotypic data of two types of *E. breviscapus*, association results and expression pattern of the candidate gene for five strong peaks on chromosome 3–6. (a) Phenotype data of two completely different types of *E. breviscapus*, which are the multileaf late flower type (left) and the sparse leaf early flower type (right). (b) Phenotypic distribution of three traits for 99 collected samples. (c) Manhattan plots of selected region and traits by SNP‐GWAS (green dots) and Indel‐GWAS (orange dots). Candidate gene close to the strong signals were highlighted by arrows. (d) Expression pattern of six candidate genes in four tissues. F, L, R and S referred to flower, leaf, root, and stem, respectively. The colour scale measures the Z‐score normalized FPKM value (fragments per kilobase of transcript per million fragments mapped) [Colour figure can be viewed at wileyonlinelibrary.com]

*E. breviscapus* exhibits substantial natural variations in leaf number, ranging from 20 to 200 leaves. Previous studies have found two completely different types of *E. breviscapus*, namely the multi‐leaf late flower type and the sparse leaf early flower type (Figure 4a; Song, et al., 2018). To identify candidate genes controlling leaf number and flowering time, we collected two groups of extreme individuals. One group consisted of 49 individuals with low leaf number (≤20) and early‐flowering, while another group consisted of 50 late‐flowering individuals with leaf numbers higher than 120 (Figure 4a). Statistics of phenotypes including branch number, leaf number and plant weight were performed (Figure 4b and Figure S6; Table S15). The phenotypic distribution showed the three traits in multileaf and sparse leaf groups were obviously distinct, especially leaf number, which illustrated the reliability of these phenotypes to distinguish samples.

By whole‐genome resequencing of 99 *E. breviscapus* individuals, we generated a total of 1640.77 Gb of clean reads, representing an average sequencing depth of 11.58× (Table S16). A total of 4,269,673 high‐quality SNPs were obtained using the updated genome as the reference for SNP calling. Among these, 4,255,459 SNPs were distributed across nine pseudo‐chromosomes, with an average density of 3 SNPs/Kb over all chromosomes. The number of SNPs on each chromosome is presented in Table S17. Chromosome 1 (925,326 SNPs) showed the highest number of SNPs, whereas the lowest number of SNPs was observed on chromosome 9 (310,510 SNPs) (Table S17). As for the SNP density, chromosome 8 had the highest density value close to 3.28 SNP/Kb, while the lowest one of 2.87 was observed on chromosome 2 (Table S17). Among the identified SNPs, 3,263,388 were found within intergenic regions, while 447,772 were found within intronic regions and 171,835 SNPs were observed within the CDS. A total of 65,869 SNPs in the CDS were synonymous, and 105,966 were nonsynonymous (Table S17). The ratio of the number of nonsynonymous to synonymous SNPs was 1.61, which was higher than that of Arabidopsis (0.83) and *O. sativa* (1.29; Clark et al., 2007; Xu et al., 2012). Additionally, a total of 1,646,738 Indels were identified on nine chromosomes, while the most (365,302) were found on chromosome 1 and the least on chromosome 9 (104,774) with an average density of 1.16 Indels per kilobase pair (Table S17).

Based on the whole‐genome SNPs, population genetic analysis was conducted. The phylogenic tree showed no clear separation of low and high leaf number group (Figure S7a), whereas the PC2 separated all 99 individuals to two clusters but two groups of high/low leaf number were mixed together (Figure S7b). By SNP‐ and Indel‐level GWAS on three traits, 54 SNPs and 25 Indels showed association with branch number, nine SNPs and five Indels with plant weight while eight SNPs and no Indels correlated with leaf number (Figure S8). When counting the significant markers on each chromosome, chromosome 5 (27 SNPs and 13 Indels) and 6 (20 SNPs) had the height number, followed by chromosome 4 (9 SNPs and 7 Indels) and 3 (5 SNPs and 5 Indels) (Table S18). As for each trait, branch number showed the most strong peaks, especially at the loci of 152,417,106 bp on chromosome 4 (Indel *p*‐value of 9.67E‐9), 8,346,064 bp on chromosome 5 (SNP *p*‐value of 1.19E‐8) and 105,039,479 bp on chromosome 6 (SNP *p*‐value of 3.70E‐8). While for leaf number and plant weight, the strongest peak were identified on 135,967,818 bp on chromosome 5 (SNP *p*‐value of 3.86E‐7) and 135,881,643 on chromosome 3 (Indel *p*‐value of 8.62E‐8), respectively. By looking deep into genes close to those peaks, six candidate genes were identified, which were *evm.model.ctg208.1*, *evm.model.ctg2378.1*, *evm.model.ctg2956.4*, *evm.model.ctg566.11*, *evm.model.ctg566.17*, and *evm.model.ctg2581.6* (Figure 4c). They were annotated as indole‐3‐pyruvate monooxygenase YUCCA2 (YUC2), zeatin *O*‐glucosyltransferase (ZOG), serine carboxypeptidase‐like 18 (SCPL18), serine/threonine‐protein phosphatase (PP1), late embryogenesis abundant protein (LEA), and F‐box protein (FBP), respectively (Table S18). Further expression patterns of these candidate genes in roots, stems, leaves and flowers indicated that *YUC2*, *PP1*, *and FBP* were expressed highest in the leaves, *ZOG* and *SCPL18* had higher expression in the roots than other three tissues, and *LEA* was expressed low in four tissues (Figure 4d).

## 4 DISCUSSION

As a self‐incompatible species in Compositae, *E. breviscapus* has a high heterozygosity genome of approximately 2.04% in the *17‐mer* analysis. Similarly, another self‐incompatible species in Compositae, *A. annua* also has a high heterozygosity of 1.0%–1.5% (Shen et al., 2018). It is a huge challenge to assemble a complex diploid genome using only short‐read sequencing (Illumina) as demonstrated by the previously published *E. breviscapus* genome (Yang et al., 2017). Nonetheless, the PacBio RSII sequencing platform generating much longer reads, greatly facilitates the sequence assembly and enhances the assembly quality (Shen et al., 2018). Here, an updated *E. breviscapus* genome was achieved by combining Illumina paired‐end sequencing data, PacBio RSII sequencing data and Hi‐C data. The new genome assembly increased in size from 1.2 Gb to 1.43 Gb, which was closer to the estimated size of 1.52 Gb by flow cytometry (Yang et al., 2017). The new genome assembly was anchored and oriented onto nine pseudo‐chromosomes, and showed significantly longer scaffold N50 and contig N50, more protein‐coding genes and higher completeness of the genome assembly compared to the previous version (Table 1). In Asteraceae, the genomes of sunflower, sweet wormwood and chrysanthemum have been released (Badouin et al., 2017; Shen et al., 2018; Song, et al., 2018). Compared with other species, both contig N50 and scaffold N50 of *E. breviscapus* are significantly longer than those of *A. annua* and chrysanthemum, though shorter than those of sunflower (Table 1). Additionally, *E. breviscapus* is the third species in Asteracea whose genome has been assembled to chromosome level, while sunflower is the first one that has been anchored onto 17 pseudo‐chromosomes (Badouin et al., 2017). These results pave the way for further genome study on *E. breviscapus* as well as Compositae in future.

*E. breviscapus* has been domesticated and selected from altitudinal gradients (1,500–2,800 m) for a long time, which shows morphological adaptation of rosette leaves and the accumulation of flavonoids in leaves. Previous studies found that the content of total flavonoids in *E. breviscapus* was positively correlated with altitude, and the correlation coefficient was 0.91999 *(p* < .001; Su et al., 2001). Our recent investigation on *E. breviscapus* at different altitudes and geographical distribution also found that the content of scutellarin in *E. breviscapus* leaves was the highest among the total flavonoids, accounting for 60%–85% (Table S20). In order to verify whether this is related to the evolution and selection of the genome, we analysed the number of gene families encoding key enzymes involved in scutellarin biosynthesis, and found that the number of downstream genes, including FSⅡ, F6H and F7GAT were significantly expanded in *E. breviscapus*, which may be the genetic basis of high content of scutellarin in *E. breviscapus*.

We previously decoded the biosynthetic pathway of breviscapine and produced scutellarin and apigenin‐7‐*O*‐glucuronide with contents reaching to 108 and 185 mg/L, respectively, by engineered yeast (Liu et al., 2018). However, scutellarin is the most abundant component in *E. breviscapus*, while the content of apigenin‐7‐*O*‐glucuronide is very low and almost undetectable. We speculated that this may be due to the poor quality of previously published *E. breviscapus* genome assembly, which lead to the incomplete capture of candidate genes and was unable to effectively convert scutellarein to scutellarin. Excitedly, we obtained two candidate genes belonging to subfamily UGT88X and one belonging to UGT88F by reanalysing the updated genome (Figure S4b). Also, more candidate genes involved in the apigenin biosynthesis were captured. Further transgenic test of *PAL, C4H, 4Cl, CHS, CHI*, and *FS II* indicated that the expression level and scutellarin contents significantly increased in the transgenic *E. breviscapus* plants compared to WT, confirming the involvement of six enzymes in apigenin biosynthesis (Figure S5c,d).

Transcriptomic analysis of *E. breviscapus* roots, stems, leaves and flowers showed that most genes involved in scutellarin biosynthesis had the highest expression in leaves, while key enzymes involved in CQA biosynthesis exhibited the highest expression levels in roots (Figure 3b). This is consistent with the fact that scutellarin is abundant in leaf and CQAs is abundant in root (2013. Those genes that locate in downstream of luteolin, kaempferol, quercetin, and hesperetin biosynthesis were differentially expressed in the roots, stems, leaves, and flowers (Figure 3b), which coincides with the finding that diverse flavonoids in *E. breviscapus* are widely distributed in different organs (Zhu et al., 2012). In addition, plant hormone treatment resulted in the upregulation of the most genes involved in flavonoids biosynthesis (Figure 3c). Previous reports have been demonstrated that methyl jasmonates (MeJA), ABA, SA and GA regulate secondary metabolism including flavonoids biosynthesis (Li et al., 2014; Mai et al., 2014; Xiao et al., 2009). Also it showed MeJA could promote scutellarin biosynthesis in *E. breviscapus* (Chen et al., 2015). Thus, plant hormone treatment is useful for screening candidate genes involved in various metabolic pathways. The genome and transcriptome data provided abundant genetic information for improving scutellarin yield from optimizing precursor genes and F7GAT.

Recent advances in high‐throughput sequencing technologies have enabled rapid and accurate resequencing of a large number of genomes (Wu et al., 2019; Yano et al., 2016). Meanwhile, the wide adoption of GWAS allows the identification of genes associated with agronomic traits in crop species (Cao et al., 2016; Huang et al., 2010). For example, Huang et al. (2012) have identified loci associated with flowering time and grain yield traits using GWAS in rice. In maize, the genetic architecture of oil biosynthesis was dissected by GWAS (Li, et al., 2013). In this study, phenotypic data indicated that for most individuals, the higher branches number, the more leaves (Table S15). Furthermore, leaf number was positively correlated with plant weight, showing leaf weight largely contributes to plant weight (45% of dry weight) (Table S15). Based on GWAS analysis of 99 resequenced individuals, the candidate genes significantly associated with the branch number, leaf number and plant weight of *E. breviscapus* were *YUC2*, *ZOG*, *SCPL18*, *PP1*, *LEA*, and *FBP* genes (Figure 4b). Among them, auxin biosynthesis by the YUC2 controls the formation of rosette leaves in *A. thaliana* (Cheng et al., 2007). SCP regulates brassinosteroids signaling that affects leaf shape, delayed flowering, and senescence in *A. thaliana* (Li et al., 2001). In plants, FBPs influence a variety of biological processes, such as leaf senescence, branching, flowering, self‐incompatibility, and responses to biotic and abiotic stresses (Yang et al., 2008). Considering the candidate gene functions and the significance of GWAS signals, *YUC2*, *SCPL18* and *FBP* may be the most possible candidate genes involved in the leaf development and flowering of *E. breviscapus*. These results provide a substantial genetic basis for genome‐assisted breeding in *E. breviscapus*.

In conclusion, among 10,000 medicinal plants, *E. breviscapus* is the only one that meets all of the following criteria: (a) Active ingredients have been decided, and the curative effects are clear. (b) There is an efficient transgenic system which provides a good technical platform for functional gene validation and gene editing. (c) De novo biosynthesis of scutellarin in engineered yeast has been accomplished (Liu et al., 2018). (d) There are also small amount of other flavonoids in *E. breviscapus* such as luteolin, kaempferol, quercetin and hesperetin (Chu et al., 2005), making it as an ideal material for elucidating the biosynthesis of flavonoids. Therefore, *E. breviscapus* is an ideal model medicinal plant in traditional Chinese medicine research, and updated genome assembly and identified candidate genes are definitely helpful for further improvement and utilization of this medicinal herb.

## AUTHOR CONTRIBUTIONS

S.Y., and Y.D. conceived the study. M.C., and M.Y. collected and grew the plant material. R.L., Y.Z., D.L., J.Y., and G.C. performed experiments. S.D., Y.L., W.F., and H.S. assembled the genome. X.D., S.H., G.Z., G.L., Y.Z., W.C., and Y.D. annotated and analysed the genome. S.H., and X.D. wrote the manuscript. All authors read and approved the final manuscript.

## Supporting information

Table of Contents

Fig S1‐S8

Table S1‐S20

## Data Availability

The whole genome sequencing and assembly have been deposited at the Sequence Read Archive (SRA) under Bioproject PRJNA525743. Transcriptome and resequencing sequence reads have been deposited under PRJNA637961 and PRJNA525744, respectively.

## References

[men13257-bib-0001] Altschul, S. F. , Gish, W. , Miller, W. , Myers, E. W. , & Lipman, D. J. (1990). Basic local alignment search tool. Journal of Molecular Biology, 215, 403–410. 10.1016/S0022-2836(05)80360-2 2231712

[men13257-bib-0002] Badouin, H. , Gouzy, J. , Grassa, C. J. , Murat, F. , Staton, S. E. , Cottret, L. , Lelandais‐Brière, C. , Owens, G. L. , Carrère, S. , Mayjonade, B. , Legrand, L. , Gill, N. , Kane, N. C. , Bowers, J. E. , Hubner, S. , Bellec, A. , Bérard, A. , Bergès, H. , Blanchet, N. , … Langlade, N. B. (2017). The sunflower genome provides insights into oil metabolism, flowering and Asterid evolution. Nature, 546, 148–152. 10.1038/nature22380 28538728

[men13257-bib-0003] Benson, G. (1999). Tandem repeats finder: A program to analyze DNA sequences. Nucleic Acids Research, 27, 573–580. 10.1093/nar/27.2.573 9862982 PMC148217

[men13257-bib-0004] Bolger, A. M. , Lohse, M. , & Usadel, B. (2014). Trimmomatic: A flexible trimmer for Illumina sequence data. Bioinformatics, 30, 2114–2120. 10.1093/bioinformatics/btu170 24695404 PMC4103590

[men13257-bib-0005] Bowers, J. E. , Pearl, S. A. , & Burke, J. M. (2016). Genetic mapping of millions of SNPs in safflower (*Carthamus tinctorius* L.) via whole‐genome resequencing. G3: Genes. Genomes, Genetics, 6, 2203–2211. 27226165 10.1534/g3.115.026690 PMC4938673

[men13257-bib-0006] Browning, B. L. , & Browning, S. R. (2016). Genotype Imputation with Millions of Reference Samples. American Journal of Human Genetics, 98, 116–126. 10.1016/j.ajhg.2015.11.020 26748515 PMC4716681

[men13257-bib-0007] Burge, S. W. , Daub, J. , Eberhardt, R. , Tate, J. , Barquist, L. , Nawrocki, E. P. , Eddy, S. R. , Gardner, P. P. , & Bateman, A. (2012). Rfam 11.0: 10 years of RNA families. Nucleic Acids Research, 41, D226–D232. 23125362 10.1093/nar/gks1005 PMC3531072

[men13257-bib-0008] Burton, J. N. , Adey, A. , Patwardhan, R. P. , Qiu, R. , Kitzman, J. O. , & Shendure, J. (2013). Chromosome‐scale scaffolding of de novo genome assemblies based on chromatin interactions. Nature Biotechnology, 31, 1119 10.1038/nbt.2727 PMC4117202 24185095

[men13257-bib-0009] Cai, Y. , González, J. V. , Liu, Z. , & Huang, T. (2014). Computational systems biology methods in molecular biology, chemistry biology, molecular biomedicine, and biopharmacy. BioMed Research International, 2014, 746814 10.1155/2014/746814 24812630 PMC4000946

[men13257-bib-0010] Cao, K. E. , Zhou, Z. , Wang, Q. I. , Guo, J. , Zhao, P. , Zhu, G. , Fang, W. , Chen, C. , Wang, X. , Wang, X. , Tian, Z. , & Wang, L. (2016). Genome‐wide association study of 12 agronomic traits in peach. Nature Communications, 7, 13246 10.1038/ncomms13246 PMC5105138 27824331

[men13257-bib-0011] Chen, J. , Tang, X. , Ren, C. , Wei, B. , Wu, Y. , Wu, Q. , & Pei, J. (2018). Full‐length transcriptome sequences and the identification of putative genes for flavonoid biosynthesis in safflower. BMC Genomics, 19, 548 10.1186/s12864-018-4946-9 30041604 PMC6057038

[men13257-bib-0012] Chen, J. , Yu, J. , Ge, L. , Wang, H. , Berbel, A. , Liu, Y. , Chen, Y. , Li, G. , Tadege, M. , Wen, J. , Cosson, V. , Mysore, K. S. , Ratet, P. , Madueno, F. , Bai, G. , & Chen, R. (2010). Control of dissected leaf morphology by a Cys (2) His (2) zinc finger transcription factor in the model legume *Medicago truncatula* . Proceedings of the National Academy of Sciences, 107, 10754–10759. 10.1073/pnas.1003954107 PMC2890821 20498057

[men13257-bib-0013] Chen, R.‐B. , Liu, J.‐H. , Xiao, Y. , Zhang, F. , Chen, J.‐F. , Ji, Q. , Tan, H.‐X. , Huang, X. , Feng, H. , Huang, B.‐K. , Chen, W.‐S. , & Zhang, L. (2015). Deep sequencing reveals the effect of MeJA on scutellarin biosynthesis in *Erigeron breviscapus* . PLoS One, 10, e0143881 10.1371/journal.pone.0143881 26656917 PMC4687647

[men13257-bib-0014] Cheng, Y. , Dai, X. , & Zhao, Y. (2007). Auxin Synthesized by the YUCCA Flavin Monooxygenases Is Essential for Embryogenesis and Leaf Formation in Arabidopsis. The Plant Cell, 19, 2430–2439. 17704214 10.1105/tpc.107.053009 PMC2002601

[men13257-bib-0015] Chinese Pharmacopoeia Commission (2015). Pharmacopoeia of the People's Republic of China. China Medical Science and Technology Press part 1, 138, 379, 848.

[men13257-bib-0016] Chu, Q. , Wu, T. , Fu, L. , & Ye, J. (2005). Simultaneous determination of active ingredients in Erigeron breviscapus (Vant.) Hand‐Mazz. by capillary electrophoresis with electrochemical detection. Journal of Pharmaceutical and Biomedical Analysis, 37, 535–541. 15740914 10.1016/j.jpba.2004.11.018

[men13257-bib-0017] Clark, R. M. , Schweikert, G. , Toomajian, C. , Ossowski, S. , Zeller, G. , Shinn, P. , Warthmann, N. , Hu, T. T. , Fu, G. , Hinds, D. A. , Chen, H. , Frazer, K. A. , Huson, D. H. , Scholkopf, B. , Nordborg, M. , Ratsch, G. , Ecker, J. R. , & Weigel, D. (2007). Common sequence polymorphisms shaping genetic diversity in *Arabidopsis thaliana* . Science, 317, 338–342. 10.1126/science.1138632 17641193

[men13257-bib-0018] Danecek, P. , Auton, A. , Abecasis, G. , Albers, C. A. , Banks, E. , DePristo, M. A. , Handsaker, R. E. , Lunter, G. , Marth, G. T. , Sherry, S. T. , McVean, G. , & Durbin, R. (2011). The variant call format and VCFtools. Bioinformatics, 27, 2156–2158. 10.1093/bioinformatics/btr330 21653522 PMC3137218

[men13257-bib-0019] De Bie, T. , Cristianini, N. , Demuth, J. P. , & Hahn, M. W. (2006). CAFE: A computational tool for the study of gene family evolution. Bioinformatics, 22, 1269–1271. 10.1093/bioinformatics/btl097 16543274

[men13257-bib-0020] Dimmer, E. C. , Huntley, R. P. , Alam‐Faruque, Y. , Sawford, T. , O'donovan, C. , Martin, M. J. , Bely, B. , Browne, P. , Mun Chan, W. , Eberhardt, R. , & Gardner, M. (2011). The UniProt‐GO annotation database in 2011. Nucleic Acids Research, 40, D565–D570. 22123736 10.1093/nar/gkr1048 PMC3245010

[men13257-bib-0021] Edgar, R. C. (2004). MUSCLE: Multiple sequence alignment with high accuracy and high throughput. Nucleic Acids Research, 32, 1792–1797. 10.1093/nar/gkh340 15034147 PMC390337

[men13257-bib-0022] Emanuelli, F. , Lorenzi, S. , Grzeskowiak, L. , Catalano, V. , Stefanini, M. , Troggio, M. , Myles, S. , Martinez‐Zapater, J. M. , Zyprian, E. , Moreira, F. M. , & Grando, M. S. (2013). Genetic diversity and population structure assessed by SSR and SNP markers in a large germplasm collection of grape. BMC Plant Biology, 13, 39 10.1186/1471-2229-13-39 23497049 PMC3610244

[men13257-bib-0023] Favaro, R. , Pinyopich, A. , Battaglia, R. , Kooiker, M. , Borghi, L. , Ditta, G. , Yanofsky, M. F. , Kater, M. M. , & Colombo, L. (2003). MADS‐box protein complexes control carpel and ovule development in Arabidopsis. The Plant Cell, 15, 2603–2611. 10.1105/tpc.015123 14555696 PMC280564

[men13257-bib-0024] Finn, R. D. , Clements, J. , & Eddy, S. R. (2011). HMMER web server: Interactive sequence similarity searching. Nucleic Acids Research, 39, W29–W37. 10.1093/nar/gkr367 21593126 PMC3125773

[men13257-bib-0066] Finn, R. D. , Tate, J. , Mistry, J. , Coggill, P. C. , Sammut, S. J. , Hotz, H.‐R. , Ceric, C. , Forslund, K. , Eddy, S.R. , Sonnhammer, E. L. L. , & Bateman, A. (2011). The Pfam protein families database. Nucleic Acids Research, 40, D290–D301. 22127870 10.1093/nar/gkr1065 PMC3245129

[men13257-bib-0025] Gardner, P. P. , Daub, J. , Tate, J. , Moore, B. L. , Osuch, I. H. , Griffiths‐Jones, S. , Finn, R. D. , Nawrocki, E. P. , Kolbe, D. L. , Eddy, S. R. , & Bateman, A. (2010). Rfam: Wikipedia, clans and the “decimal” release. Nucleic Acids Research, 39, D141–D145. 21062808 10.1093/nar/gkq1129 PMC3013711

[men13257-bib-0026] Haas, B. J. , Delcher, A. L. , Mount, S. M. , Wortman, J. R. , Smith, R. K. Jr , Hannick, L. I. , & Salzberg, S. L. (2003). Improving the Arabidopsis genome annotation using maximal transcript alignment assemblies. Nucleic Acids Research, 31, 5654–5666. 10.1093/nar/gkg770 14500829 PMC206470

[men13257-bib-0027] Haas, B. J. , Salzberg, S. L. , Zhu, W. , Pertea, M. , Allen, J. E. , Orvis, J. , White, O. , Buell, C. R. , & Wortman, J. R. (2008). Automated eukaryotic gene structure annotation using EVidenceModeler and the Program to Assemble Spliced Alignments. Genome Biology, 9, 1 10.1186/gb-2008-9-1-r7 PMC2395244 18190707

[men13257-bib-0028] Hackl, T. , Hedrich, R. , Schultz, J. , & Forster, F. (2014). proovread: Large‐scale high‐accuracy PacBio correction through iterative short read consensus. Bioinformatics, 30, 3004–3011. 10.1093/bioinformatics/btu392 25015988 PMC4609002

[men13257-bib-0029] Hirakawa, H. , Sumitomo, K. , Hisamatsu, T. , Nagano, S. , Shirasawa, K. , Higuchi, Y. , Kusaba, M. , Koshioka, M. , Nakano, Y. , Yagi, M. , Yamaguchi, H. , Taniguchi, K. , Nakano, M. , & Isobe, S. N. (2019). *De novo* whole‐genome assembly in *Chrysanthemum seticuspe*, a model species of Chrysanthemums, and its application to genetic and gene discovery analysis. DNA Research, 26, 195–203. 10.1093/dnares/dsy048 30689773 PMC6589549

[men13257-bib-0030] Hirotani, M. , Kuroda, R. , Suzuki, H. , & Yoshikawa, T. (2000). Cloning and expression of UDP‐glucose: Flavonoid 7‐*O*‐glucosyltransferase from hairy root cultures of *Scutellaria baicalensis* . Planta, 210, 1006–1013. 10.1007/PL00008158 10872235

[men13257-bib-0031] Huang, X. , Wei, X. , Sang, T. , Zhao, Q. , Feng, Q. I. , Zhao, Y. , Li, C. , Zhu, C. , Lu, T. , Zhang, Z. , Li, M. , Fan, D. , Guo, Y. , Wang, A. , Wang, L. U. , Deng, L. , Li, W. , Lu, Y. , Weng, Q. , … Han, B. (2010). Genome‐wide association studies of 14 agronomic traits in rice landraces. Nature Genetics, 42, 961–967. 10.1038/ng.695 20972439

[men13257-bib-0032] Huang, X. , Zhao, Y. , Wei, X. , Li, C. , Wang, A. , Zhao, Q. , Li, W. , Guo, Y. , Deng, L. , Zhu, C. , Fan, D. , Lu, Y. , Weng, Q. , Liu, K. , Zhou, T. , Jing, Y. , Si, L. , Dong, G. , Huang, T. , … Han, B. (2012). Genome‐wide association study of flowering time and grain yield traits in a worldwide collection of rice germplasm. Nature Genetics, 44, 32 10.1038/ng.1018 22138690

[men13257-bib-0033] Hunter, S. , Jones, P. , Mitchell, A. , Apweiler, R. , Attwood, T. K. , Bateman, A. , Bernard, T. , Binns, D. , Bork, P. , Burge, S. , & De Castro, E. (2011). InterPro in 2011: New developments in the family and domain prediction database. Nucleic Acids Research, 40, D306–D312. 22096229 10.1093/nar/gkr948 PMC3245097

[men13257-bib-0034] Jaakola, L. , & Hohtola, A. (2010). Effect of latitude on flavonoid biosynthesis in plants. Plant Cell and Environment, 33, 1239–1247. 10.1111/j.1365-3040.2010.02154.x 20374534

[men13257-bib-0035] Jiang, N.‐H. , Zhang, G.‐H. , Zhang, J.‐J. , Shu, L.‐P. , Zhang, W. , Long, G.‐Q. , Liu, T. , Meng, Z.‐G. , Chen, J.‐W. , & Yang, S.‐C. (2014). Analysis of the transcriptome of *Erigeron breviscapus* uncovers putative scutellarin and chlorogenic acids biosynthetic genes and genetic markers. PLoS One, 9, e100357 10.1371/journal.pone.0100357 24956277 PMC4067309

[men13257-bib-0036] Jurka, J. , Kapitonov, V. V. , Pavlicek, A. , Klonowski, P. , Kohany, O. , & Walichiewicz, J. (2005). Repbase Update, a database of eukaryotic repetitive elements. Cytogenetic and Genome Research, 110, 462–467. 10.1159/000084979 16093699

[men13257-bib-0037] Kanehisa, M. , & Goto, S. (2000). KEGG: Kyoto encyclopedia of genes and genomes. Nucleic Acids Research, 28, 27–30. 10.1093/nar/28.1.27 10592173 PMC102409

[men13257-bib-0038] Korf, I. (2004). Gene finding in novel genomes. BMC Bioinformatics, 5, 59. 15144565 10.1186/1471-2105-5-59 PMC421630

[men13257-bib-0039] Kumar, S. , Stecher, G. , & Tamura, K. (2016). MEGA7: Molecular evolutionary genetics analysis version 7.0 for bigger datasets. Molecular Biology and Evolution, 33, 1870–1874. 10.1093/molbev/msw054 27004904 PMC8210823

[men13257-bib-0040] Legrand, G. , Delporte, M. , Khelifi, C. , Harant, A. , Vuylsteker, C. , Mörchen, M. , Hance, P. , Hilbert, J.‐L. , & Gagneul, D. (2016). Identification and characterization of five BAHD acyltransferases involved in hydroxycinnamoyl ester metabolism in chicory. Frontiers in Plant Science, 7, 741 10.3389/fpls.2016.00741 27375627 PMC4893494

[men13257-bib-0041] Letunic, I. , & Bork, P. (2019). Interactive Tree Of Life (iTOL) v4: Recent updates and new developments. Nucleic Acids Research, 47, 256–259. 10.1093/nar/gkz239 PMC6602468 30931475

[men13257-bib-0043] Li, H. , & Durbin, R. (2009). Fast and accurate short read alignment with Burrows‐Wheeler transform. Bioinformatics, 25, 1754–1760. 10.1093/bioinformatics/btp324 19451168 PMC2705234

[men13257-bib-0044] Li, H. , Handsaker, B. , Wysoker, A. , Fennell, T. , Ruan, J. , Homer, N. , Marth, G. , Abecasis, G. , & Durbin, R. (2009). The sequence alignment/map format and SAMtools. Bioinformatics, 25, 2078–2079. 10.1093/bioinformatics/btp352 19505943 PMC2723002

[men13257-bib-0045] Li, H. , Peng, Z. , Yang, X. , Wang, W. , Fu, J. , Wang, J. , Han, Y. , Chai, Y. , Guo, T. , Yang, N. , Liu, J. , Warburton, M. L. , Cheng, Y. , Hao, X. , Zhang, P. , Zhao, J. , Liu, Y. , Wang, G. , Li, J. , & Yan, J. (2013). Genome‐wide association study dissects the genetic architecture of oil biosynthesis in maize kernels. Nature Genetics, 45, 43–50. 10.1038/ng.2484 23242369

[men13257-bib-0046] Li, J. , Lease, K. A. , Tax, F. E. , & Walker, J. C. (2001). BRS1, a serine carboxypeptidase, regulates BRI1 signaling in *Arabidopsis thaliana* . Proceedings of the National Academy of Sciences of the United States of America, 98, 5916–5921. 10.1073/pnas.091065998 11320207 PMC33313

[men13257-bib-0047] Li, L. , Stoeckert, C. J. , & Roos, D. S. (2003). OrthoMCL: Identification of ortholog groups for eukaryotic genomes. Genome Research, 13, 2178–2189. 10.1101/gr.1224503 12952885 PMC403725

[men13257-bib-0048] Li, T. , Jia, K. P. , Lian, H. L. , Yang, X. , Li, L. , & Yang, H. Q. (2014). Jasmonic acid enhancement of anthocyanin accumulation is dependent on phytochrome A signaling pathway under far‐red light in Arabidopsis. Biochemical and Biophysical Research Communications, 454, 78–83. 10.1016/j.bbrc.2014.10.059 25450360

[men13257-bib-0049] Li, X. B. , Wang, R. B. , Shen, Y. , Meng, Z. G. , Chen, J. W. , Yang, J. W. , & Yang, S. C. (2013). Simultaneous determination of chlorogenic acid, scutellarin, 3, 5‐dicaffeoylquinic acid, 4, 5‐dicaffeoylquinic acid in different parts of *Erigeron breviscapus* by high‐performance liquid chromatography. China Journal of Chinese Materia Medica, 38, 2237–2240. 24199546

[men13257-bib-0050] Liu, X. , Cheng, J. , Zhang, G. , Ding, W. , Duan, L. , Yang, J. , Kui, L. , Cheng, X. , Ruan, J. , Fan, W. , Chen, J. , Long, G. , Zhao, Y. , Cai, J. , Wang, W. , Ma, Y. , Dong, Y. , Yang, S. , & Jiang, H. (2018). Engineering yeast for the production of breviscapine by genomic analysis and synthetic biology approaches. Nature Communications, 9, 448 10.1038/s41467-018-02883-z PMC5792594 29386648

[men13257-bib-0051] Lowe, T. M. , & Eddy, S. R. (1997). tRNAscan‐SE: A program for improved detection of transfer RNA genes in genomic sequence. Nucleic Acids Research, 25, 955–964. 10.1093/nar/25.5.955 9023104 PMC146525

[men13257-bib-0052] Mai, V. C. , Drzewiecka, K. , Jeleń, H. , Narożna, D. , Rucińska‐Sobkowiak, R. , Kęsy, J. , Floryszak‐Wieczorek, J. , Gabryś, B. , & Morkunas, I. (2014). Differential induction of *Pisum sativum* defense signaling molecules in response to pea aphid infestation. Plant Science, 221–222, 1–12. 10.1016/j.plantsci.2014.01.011 24656330

[men13257-bib-0053] Marçais, G. , & Kingsford, C. (2011). A fast, lock‐free approach for efficient parallel counting of occurrences of k‐mers. Bioinformatics, 27, 764–770. 10.1093/bioinformatics/btr011 21217122 PMC3051319

[men13257-bib-0054] Marchler‐Bauer, A. , Lu, S. , Anderson, J. B. , Chitsaz, F. , Derbyshire, M. K. , DeWeese‐Scott, C. , Fong, J. H. , Geer, L. Y. , Geer, R. C. , Gonzales, N. R. , & Gwadz, M. (2010). CDD: A Conserved Domain Database for the functional annotation of proteins. Nucleic Acids Research, 39, D225–D229. 21109532 10.1093/nar/gkq1189 PMC3013737

[men13257-bib-0055] McKenna, A. , Hanna, M. , Banks, E. , Sivachenko, A. , Cibulskis, K. , Kernytsky, A. , Garimella, K. , Altshuler, D. , Gabriel, S. , Daly, M. , & DePristo, M. A. (2010). The Genome Analysis Toolkit: A MapReduce framework for analyzing next‐generation DNA sequencing data. Genome Research, 20, 1297–1303. 10.1101/gr.107524.110 20644199 PMC2928508

[men13257-bib-0056] Moglia, A. , Acquadro, A. , Eljounaidi, K. , Milani, A. M. , Cagliero, C. , Rubiolo, P. , Genre, A. , Cankar, K. , Beekwilder, J. , & Comino, C. (2016). Genome‐wide identification of bahd acyltransferases and *in vivo* characterization of HQT‐like enzymes involved in caffeoylquinic acid synthesis in globe artichoke. Frontiers in Plant Science, 7, 1424 10.3389/fpls.2016.01424 27721818 PMC5033976

[men13257-bib-0057] Moglia, A. , Lanteri, S. , Comino, C. , Hill, L. , Knevitt, D. , Cagliero, C. , Rubiolo, P. , Bornemann, S. , & Martin, C. (2014). Dual catalytic activity of hydroxycinnamoyl‐coenzyme a quinate transferase from tomato allows it to moonlight in the synthesis of both mono‐ and dicaffeoylquinic acids. Plant Physiology, 166, 1777–1787. 10.1104/pp.114.251371 25301886 PMC4256858

[men13257-bib-0058] Nagashima, S. , Hirotani, M. , & Yoshikawa, T. (2000). Purification and characterization of UDP‐glucuronate: Baicalein 7‐*O*‐glucuronosyltransferase from *Scutellaria baicalensis* Georgi. cell suspension cultures. Phytochemistry, 53, 533–538. 10.1016/S0031-9422(99)00593-2 10724177

[men13257-bib-0059] Nawrocki, E. P. , Kolbe, D. L. , & Eddy, S. R. (2009). Infernal 1.0: Inference of RNA alignments. Bioinformatics, 25, 1335–1337. 10.1093/bioinformatics/btp157 19307242 PMC2732312

[men13257-bib-0060] Noguchi, A. , Horikawa, M. , Fukui, Y. , Fukuchi‐Mizutani, M. , Iuchi‐Okada, A. , Ishiguro, M. , Kiso, Y. , Nakayama, T. , & Ono, E. (2009). Local differentiation of sugar donor specificity of flavonoid glycosyltransferase in Lamiales. The Plant Cell, 21, 1556–1572. 10.1105/tpc.108.063826 19454730 PMC2700533

[men13257-bib-0061] Ono, E. , Ruike, M. , Iwashita, T. , Nomoto, K. , & Fukui, Y. (2010). Co‐pigmentation and flavonoid glycosyltransferases in blue *Veronica persica* flowers. Phytochemistry, 71, 726–735. 10.1016/j.phytochem.2010.02.008 20223486

[men13257-bib-0062] Pandey, R. P. , Parajuli, P. , Koffas, M. A. , & Sohng, J. K. (2016). Microbial production of natural and non‐natural flavonoids: Pathway engineering, directed evolution and systems/synthetic biology. Biotechnology Advances, 34, 634–662. 10.1016/j.biotechadv.2016.02.012 26946281

[men13257-bib-0063] Patel, R. K. , & Jain, M. (2012). NGS QC Toolkit: A toolkit for quality control of next generation sequencing data. PLoS One, 7, e30619 10.1371/journal.pone.0030619 22312429 PMC3270013

[men13257-bib-0064] Peng, Y. , Lai, Z. , Lane, T. , Nageswara‐Rao, M. , Okada, M. , Jasieniuk, M. , O'Geen, H. , Kim, R. W. , Sammons, R. D. , Rieseberg, L. H. , & Stewart, C. N. (2014). *De novo* genome assembly of the economically important weed horseweed using integrated data from multiple sequencing platforms. Plant Physiology, 166, 1241–1254. 10.1104/pp.114.247668 25209985 PMC4226366

[men13257-bib-0065] Petrussa, E. , Braidot, E. , Zancani, M. , Peresson, C. , Bertolini, A. , Patui, S. , & Vianello, A. (2013). Plant Flavonoids—Biosynthesis, Transport and Involvement in Stress Responses. International Journal of Molecular Sciences, 14, 14950–14973. 10.3390/ijms140714950 23867610 PMC3742282

[men13257-bib-0067] Qiu, Q. , Zhang, G. , Ma, T. , Qian, W. , Wang, J. , Ye, Z. , Cao, C. , Hu, Q. , Kim, J. , Larkin, D. M. , Auvil, L. , Capitanu, B. , Ma, J. , Lewin, H. A. , Qian, X. , Lang, Y. , Zhou, R. , Wang, L. , Wang, K. , … Liu, J. (2012). The yak genome and adaptation to life at high altitude. Nature Genetics, 44, 946 10.1038/ng.2343 22751099

[men13257-bib-0068] Renwei, Z. , Xian'e, F. , & Laiwei, L. (2011). Modernization of Traditional Chinese Medicine Injection‐R&D of Breviscapine Injection. World Science and Technology, 6, 24.

[men13257-bib-0069] Reyes‐Chin‐Wo, S. , Wang, Z. , Yang, X. , Kozik, A. , Arikit, S. , Song, C. , Xia, L. , Froenicke, L. , Lavelle, D. O. , Truco, M.‐J. , Xia, R. , Zhu, S. , Xu, C. , Xu, H. , Xu, X. , Cox, K. , Korf, I. , Meyers, B. C. , & Michelmore, R. W. (2017). Genome assembly with in vitro proximity ligation data and whole‐genome triplication in lettuce. Nature Communications, 8, 14953 10.1038/ncomms14953 PMC5394340 28401891

[men13257-bib-0070] Scaglione, D. , Reyes‐Chin‐Wo, S. , Acquadro, A. , Froenicke, L. , Portis, E. , Beitel, C. , Tirone, M. , Mauro, R. , Monaco, A. L. , Mauromicale, G. , & Faccioli, P. (2016). The genome sequence of the outbreeding globe artichoke constructed de novo incorporating a phase‐aware low‐pass sequencing strategy of F1 progeny. Scientific Reports, 6, 19427. 26786968 10.1038/srep19427 PMC4726258

[men13257-bib-0071] Servant, N. , Varoquaux, N. , Lajoie, B. R. , Viara, E. , Chen, C.‐J. , Vert, J.‐P. , Heard, E. , Dekker, J. , & Barillot, E. (2015). HiC‐Pro: An optimized and flexible pipeline for Hi‐C data processing. Genome Biology, 16, 259 10.1186/s13059-015-0831-x 26619908 PMC4665391

[men13257-bib-0072] Shen, Q. , Zhang, L. , Liao, Z. , Wang, S. , Yan, T. , Shi, P. U. , Liu, M. , Fu, X. , Pan, Q. , Wang, Y. , Lv, Z. , Lu, X. U. , Zhang, F. , Jiang, W. , Ma, Y. , Chen, M. , Hao, X. , Li, L. , Tang, Y. , … Tang, K. (2018). The genome of *Artemisia annua* provides insight into the evolution of Asteraceae family and Artemisinin Biosynthesis. Molecular Plant, 11, 776–788. 10.1016/j.molp.2018.03.015 29703587

[men13257-bib-0073] Simao, F. A. , Waterhouse, R. M. , Ioannidis, P. , Kriventseva, E. V. , & Zdobnov, E. M. (2015). BUSCO: Assessing genome assembly and annotation completeness with single‐copy orthologs. Bioinformatics, 31, 3210–3212. 10.1093/bioinformatics/btv351 26059717

[men13257-bib-0074] Singh, B. , Kumar, A. , & Malik, A. K. (2017). Flavonoids biosynthesis in plants and its further analysis by capillary electrophoresis. Electrophoresis, 38, 820–832. 10.1002/elps.201600334 27921314

[men13257-bib-0075] Song, C. , Liu, Y. , Song, A. , Dong, G. , Zhao, H. , Sun, W. , Ramakrishnan, S. , Wang, Y. , Wang, S. , Li, T. , Niu, Y. , Jiang, J. , Dong, B. , Xia, Y. E. , Chen, S. , Hu, Z. , Chen, F. , & Chen, S. (2018). The Chrysanthemum nankingense genome provides insights into the evolution and diversification of chrysanthemum flowers and medicinal traits. Molecular Plant, 11, 1482–1491. 10.1016/j.molp.2018.10.003 30342096

[men13257-bib-0076] Song, W. , Lu, Y. , Liu, S. , Zhang, G. , & Yang, S. (2018). Correlation analysis between agronomic traits and economic characters of *Erigeron breviscapus* . Molecular Plant Breeding, 16, 7148–7158.

[men13257-bib-0077] Stanke, M. , Keller, O. , Gunduz, I. , Hayes, A. , Waack, S. , & Morgenstern, B. (2006). AUGUSTUS: Ab initio prediction of alternative transcripts. Nucleic Acids Research, 34, W435–W439. 10.1093/nar/gkl200 16845043 PMC1538822

[men13257-bib-0078] Su, W. , Lu, J. , Zhang, G. , & Wang, C. (2001). Ecological and biological analysis of total flavonoids in *Erigeron breviscapus* . Chinese Traditional and Herbal Drugs, 32, 1119–1121.

[men13257-bib-0079] Sun, X. , Zhang, Z. , Chen, C. , Wu, W. , Ren, N. , Jiang, C. , Yu, J. , Zhao, Y. , Zheng, X. , Yang, Q. , Zhang, H. , Li, J. , & Li, Z. (2018). The C‐S–A gene system regulates hull pigmentation and reveals evolution of anthocyanin biosynthesis pathway in rice. Journal of Experimental Botany, 69, 1485–1498. 10.1093/jxb/ery001 29361187 PMC5888925

[men13257-bib-0080] Tarailo‐Graovac, M. , & Chen, N. (2009). Using RepeatMasker to identify repetitive elements in genomic sequences. Current Protocols in Bioinformatics, 25, 4–10. 10.1002/0471250953.bi0410s25 19274634

[men13257-bib-0081] Trapnell, C. , Roberts, A. , Goff, L. , Pertea, G. , Kim, D. , Kelley, D. R. , Pimentel, H. , Salzberg, S. L. , Rinn, J. L. , & Pachter, L. (2012). Differential gene and transcript expression analysis of RNA‐seq experiments with TopHat and Cufflinks. Nature Protocols, 7, 562 10.1038/nprot.2012.016 22383036 PMC3334321

[men13257-bib-0082] Turner, S. D. (2014). qqman: an R package for visualizing GWAS results using Q‐Q and manhattan plots. bioRxiv.

[men13257-bib-0083] Vidic, D. , Ćavar Zeljković, S. , Dizdar, M. , & Maksimović, M. (2016). Essential oil composition and antioxidant activity of four Asteraceae species from Bosnia. Journal of Essential Oil Research, 28, 445–457. 10.1080/10412905.2016.1150216

[men13257-bib-0084] Wang, K. , Li, M. , & Hakonarson, H. (2010). ANNOVAR: Functional annotation of genetic variants from high‐throughput sequencing data. Nucleic Acids Research, 38, e164 10.1093/nar/gkq603 20601685 PMC2938201

[men13257-bib-0085] Wang, Y. , Chen, S. , & Yu, O. (2011). Metabolic engineering of flavonoids in plants and microorganisms. Applied Microbiology and Biotechnology, 91, 949–956. 10.1007/s00253-011-3449-2 21732240

[men13257-bib-0086] Waterhouse, R. M. , Seppey, M. , Simão, F. A. , Manni, M. , Ioannidis, P. , Klioutchnikov, G. , Kriventseva, E. V. , & Zdobnov, E. M. (2017). BUSCO applications from quality assessments to gene prediction and phylogenomics. Molecular Biology and Evolution, 35, 543–548. 10.1093/molbev/msx319 PMC5850278 29220515

[men13257-bib-0087] Wu, D. , Liang, Z. , Yan, T. , Xu, Y. , Xuan, L. , Tang, J. , Zhou, G. , Lohwasser, U. , Hua, S. , Wang, H. , Chen, X. , Wang, Q. , Zhu, L. E. , Maodzeka, A. , Hussain, N. , Li, Z. , Li, X. , Shamsi, I. H. , Jilani, G. , … Jiang, L. (2019). Whole‐genome resequencing of a worldwide collection of rapeseed accessions reveals the genetic basis of ecotype divergence. Molecular Plant, 12, 30–43. 10.1016/j.molp.2018.11.007 30472326

[men13257-bib-0088] Xiao, Y. , Gao, S. , Di, P. , Chen, J. , Chen, W. , & Zhang, L. (2009). Methyl jasmonate dramatically enhances the accumulation of phenolic acids in *Salvia miltiorrhiza* hairy root cultures. Physiologia Plantarum, 137, 1–9. 19570133 10.1111/j.1399-3054.2009.01257.x

[men13257-bib-0089] Xu, X. , Liu, X. , Ge, S. , Jensen, J. D. , Hu, F. Y. , Li, X. , Dong, Y. , Gutenkunst, R. N. , Fang, L. , Huang, L. , Li, J. , He, W. , Zhang, G. , Zheng, X. , Zhang, F. , Li, Y. , Yu, C. , Kristiansen, K. , Zhang, X. , … Wang, W. (2012). Resequencing 50 accessions of cultivated and wild rice yields markers for identifying agronomically important genes. Nature Biotechnology, 3, 105–157. 10.1038/nbt.2050 22158310

[men13257-bib-0090] Xu, Z. , & Wang, H. (2007). LTR_FINDER: An efficient tool for the prediction of full‐length LTR retrotransposons. Nucleic Acids Research, 35, W265–W268. 10.1093/nar/gkm286 17485477 PMC1933203

[men13257-bib-0091] Yang, J. , Lee, S. H. , Goddard, M. E. , & Visscher, P. M. (2011). GCTA: A tool for genome‐wide complex trait analysis. The American Journal of Human Genetics, 88, 76–82. 10.1016/j.ajhg.2010.11.011 21167468 PMC3014363

[men13257-bib-0092] Yang, J. , Zhang, G. , Zhang, J. , Liu, H. , Chen, W. , Wang, X. , Li, Y. , Dong, Y. , & Yang, S. (2017). Hybrid de novo genome assembly of the Chinese herbal fleabane *Erigeron breviscapus* . GigaScience, 6, 1–7. 10.1093/gigascience/gix028 PMC5449645 28431028

[men13257-bib-0093] Yang, X. , Kalluri, U. C. , Jawdy, S. , Gunter, L. E. , Yin, T. , Tschaplinski, T. J. , Weston, D. J. , Ranjan, P. , & Tuskan, G. A. (2008). The F‐box gene family is expanded in herbaceous annual plants relative to woody perennial plants. Plant Physiology, 148, 1189–1200. 10.1104/pp.108.121921 18775973 PMC2577272

[men13257-bib-0094] Yang, Z. (2007). PAML 4: Phylogenetic analysis by maximum likelihood. Molecular Biology and Evolution, 24, 1586–1591. 10.1093/molbev/msm088 17483113

[men13257-bib-0095] Yano, K. , Yamamoto, E. , Aya, K. , Takeuchi, H. , Lo, P.‐C. , Hu, L. I. , Yamasaki, M. , Yoshida, S. , Kitano, H. , Hirano, K. O. , & Matsuoka, M. (2016). Genome‐wide association study using whole‐genome sequencing rapidly identifies new genes influencing agronomic traits in rice. Nature Genetics, 48, 927–934. 10.1038/ng.3596 27322545

[men13257-bib-0096] Yuan, Y. , Wang, Z. , Jiang, C. , Wang, X. , & Huang, L. (2014). Exploiting genes and functional diversity of chlorogenic acid and luteolin biosyntheses in *Lonicera japonica* and their substitutes. Gene, 534, 408–416. 10.1016/j.gene.2012.09.051 23085319 PMC7138419

[men13257-bib-0097] Zhang, K. E. , Wang, R. , Zi, H. , Li, Y. , Cao, X. , Li, D. , Guo, L. , Tong, J. , Pan, Y. , Jiao, Y. , Liu, R. , Xiao, L. , & Liu, X. (2018). AUXIN RESPONSE FACTOR3 regulates floral meristem determinacy by repressing cytokinin biosynthesis and signaling. The Plant Cell, 30, 324–346. 10.1105/tpc.17.00705 29371438 PMC5868698

[men13257-bib-0098] Zhang, L. , Liu, C. , Lin, L. , & Chen, W. (2007). Callus Induction and Adventitious Shoot Regeneration from Petiole of *Erigeron breviscapus* . Plant Production Science, 10, 343–345.

[men13257-bib-0099] Zhang, Z. , Li, J. , Zhao, X. Q. , Wang, J. , Wong, G. K. S. , & Yu, J. (2006). KaKs_Calculator: Calculating Ka and Ks through model selection and model averaging. Genomics, Proteomics & Bioinformatics, 4, 259–263. 10.1016/S1672-0229(07)60007-2 PMC5054075 17531802

[men13257-bib-0100] Zhang, W. , Xiang, W. , Meng, H. L. , Ma, C. H. , Jiang, N. H. , Zhang, G. H. , & Yang, S. C. (2015). Transcriptomic comparison of the self‐pollinated and cross‐pollinated flowers of Erigeron breviscapus to analyze candidate self‐incompatibility‐associated genes. BMC Plant Biology, 15, 248. 26463824 10.1186/s12870-015-0627-x PMC4604739

[men13257-bib-0101] Zhou, X. , & Stephens, M. (2012). Genome‐wide efficient mixed‐model analysis for association studies. Nature Genetics, 44, 821–824. 10.1038/ng.2310 22706312 PMC3386377

[men13257-bib-0102] Zhu, F. , Yang, F. , Wang, J. , Zhang, Y. , & Chen, Y. (2012). Research Progress of Flavonoids in *Erigeron breviscapus* . Journal of Anhui Agricultural Sciences, 40, 5853–5857.

