# Supplementary material for: High quality genome of Erigeron breviscapus provides a reference for herbal plants in Asteraceae
Source: Mol Ecol Resour. 2020 Oct 22;21(1):153–69. doi: 10.1111/1755-0998.13257 (PMC7756436; doi:10.1111/1755-0998.13257)
Supplement: Supplementary file 1 — Table of Contents [file MEN-21-153-s001.docx]

**Supplemental Information for:**

**High quality genome of *Erigeron breviscapus* provides a reference for**

**herbal plants in Asteraceae**

Simei He, Xiao Dong, Guanghui Zhang, Wei Fan, Shengchang Duan, Hong Shi, Dawei Li, Rui Li, Geng Chen, Guangqiang Long, Yan Zhao, Mo Chen, Mi Yan, Jianli Yang, Yingchun Lu, Yanli Zhou, Wei Chen, Yang Dong, Shengchao Yang

**Table of Contents:**

| **Table S1. Statistics of the genome sequencing libraries.** | **Page 1** |
| --- | --- |
| **Table S2. Assembly statistics for the genome of *E. breviscapus*.** | **Page 2** |
| **Table S3. Assessing *E. breviscapus* genome and annotation completeness with BUSCO.** | **Page 3** |
| **Table S4. Statistics of repeats in the *E. breviscapus* genome.** | **Page 4** |
| **Table S5. Summary of transposable elements in the *E. breviscapus* genome assembly.** | **Page 5** |
| **Table S6. Statistics of the RNA sequencing libraries.** | **Page 6** |
| **Table S7. Summary of protein-coding gene annotation in the *E. breviscapus* genome assembly.** | **Page 7** |
| **Table S8. Summary of non-protein-coding gene annotation in the *E. breviscapus* genome assembly.** | **Page 8** |
| **Table S9. Gene family clustering analysis.** | **Page 9** |
| **Table S10. Distribution patterns of gene involved in scutellarin biosynthesis across different plants.** | **Page 10** |
| **Table S11. Positively selected genes between *E. breviscapus* and *A. annua*.** | **Page 11** |
| **Table S12. Gene involved in flavonoids and caffeoylquinic acids biosynthesis in *E. breviscapus*.** | **Page 12** |
| **Table S13. The protein sequences of UGT88.** | **Page 13** |
| **Table S14. Primers used to amplify cDNA of genes involved in flavones biosynthesis for insertion into**  **pCambia1301-35SN expression.** | **Page 14** |
| **Table S15. Phenotypic data of the three traits in the *E. breviscapus*.** | **Page 15** |
| **Table S16. Statistics of the resequencing libraries.** | **Page 16** |
| **Table S17. Summary of SNP and Indel.** | **Page 17** |
| **Table S18. Genome-wide significant association signals of 3 traits and related candidate genes.** | **Page 18** |
| **Table S19. Summary of all software parameters used in this text.** | **Page 19** |
| **Table S20. The sampling location information and the concentrations of scutellarin and total flavonoids**  **in *E. breviscapus*.** | **Page 20** |

**

Instructions for Authors:

1. use of this branded Supplemental Information template is recommended, but not mandatory
2. consolidate your Supplemental files into as few documents as possible
3. if your file of Supplemental Information is very large, create a Table of Contents
4. Your Supplemental Information will not be copy-edited. Do not leave in track-changes and other editing marks. The document will be posted "as is.”
5. Save as PDF if possible
