# Supplementary material for: High quality genome of Erigeron breviscapus provides a reference for herbal plants in Asteraceae
Source: Mol Ecol Resour. 2020 Oct 22;21(1):153–69. doi: 10.1111/1755-0998.13257 (PMC7756436; doi:10.1111/1755-0998.13257)
Supplement: Supplementary file 2 — Fig S1‐S8 [file MEN-21-153-s002.pdf]

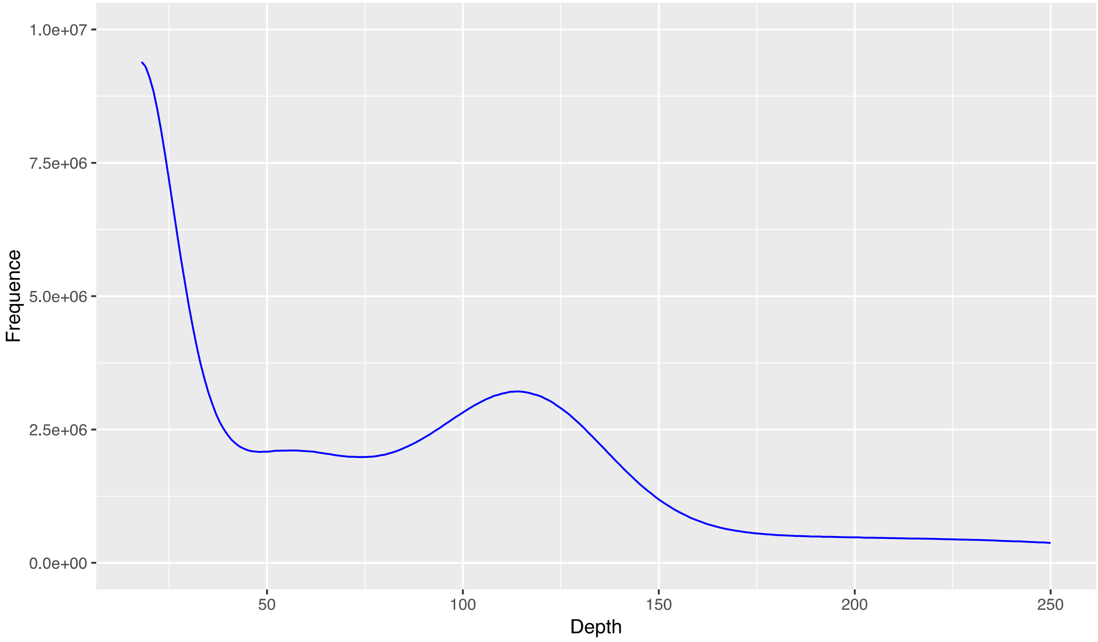

Figure S1. Frequency distribution of the *17-mer* graph.

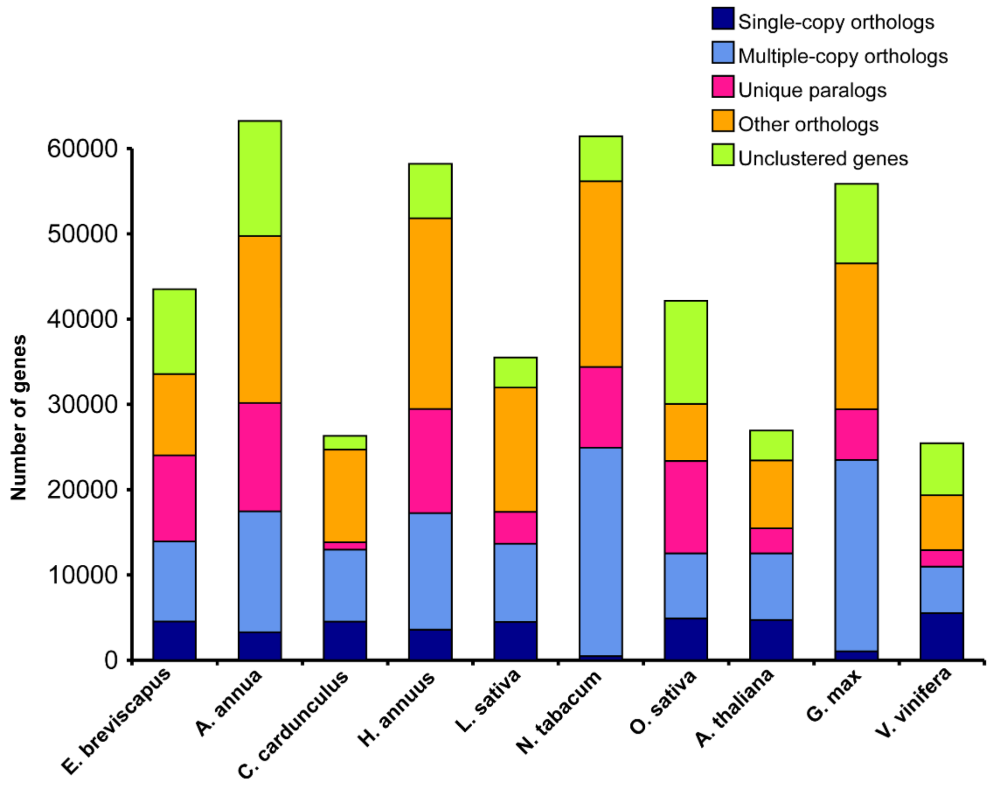

Figure S2. Ortholog clustering analysis of the protein-coding genes in the *E. breviscapus* genome.

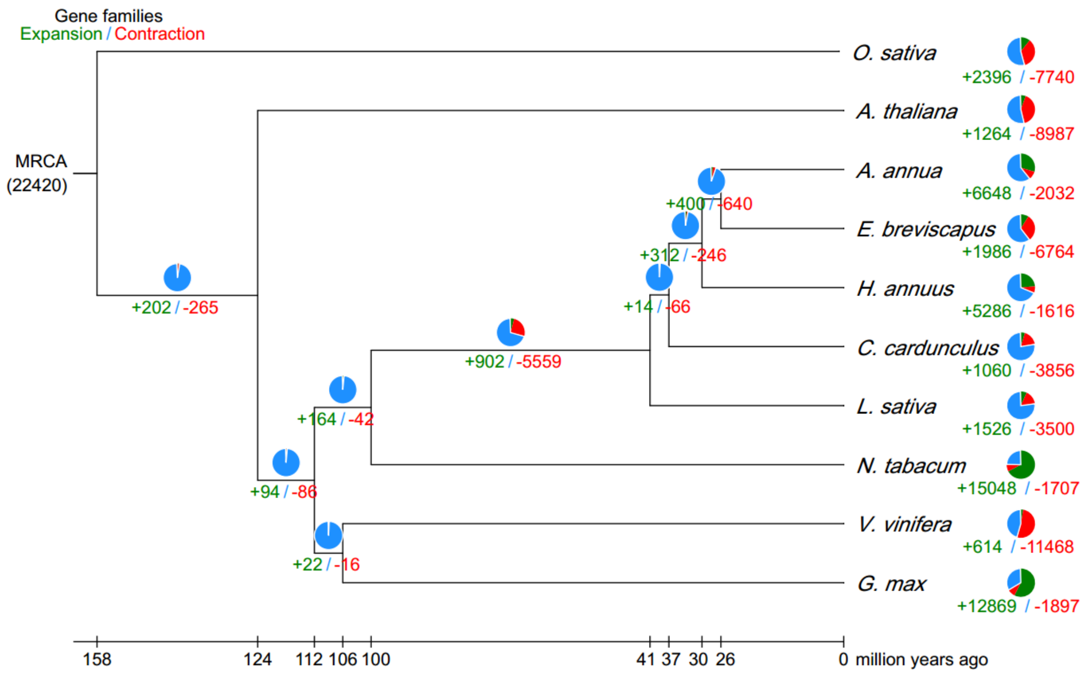

Figure S3. Gene family expansions and contractions in the *E. breviscapus*.

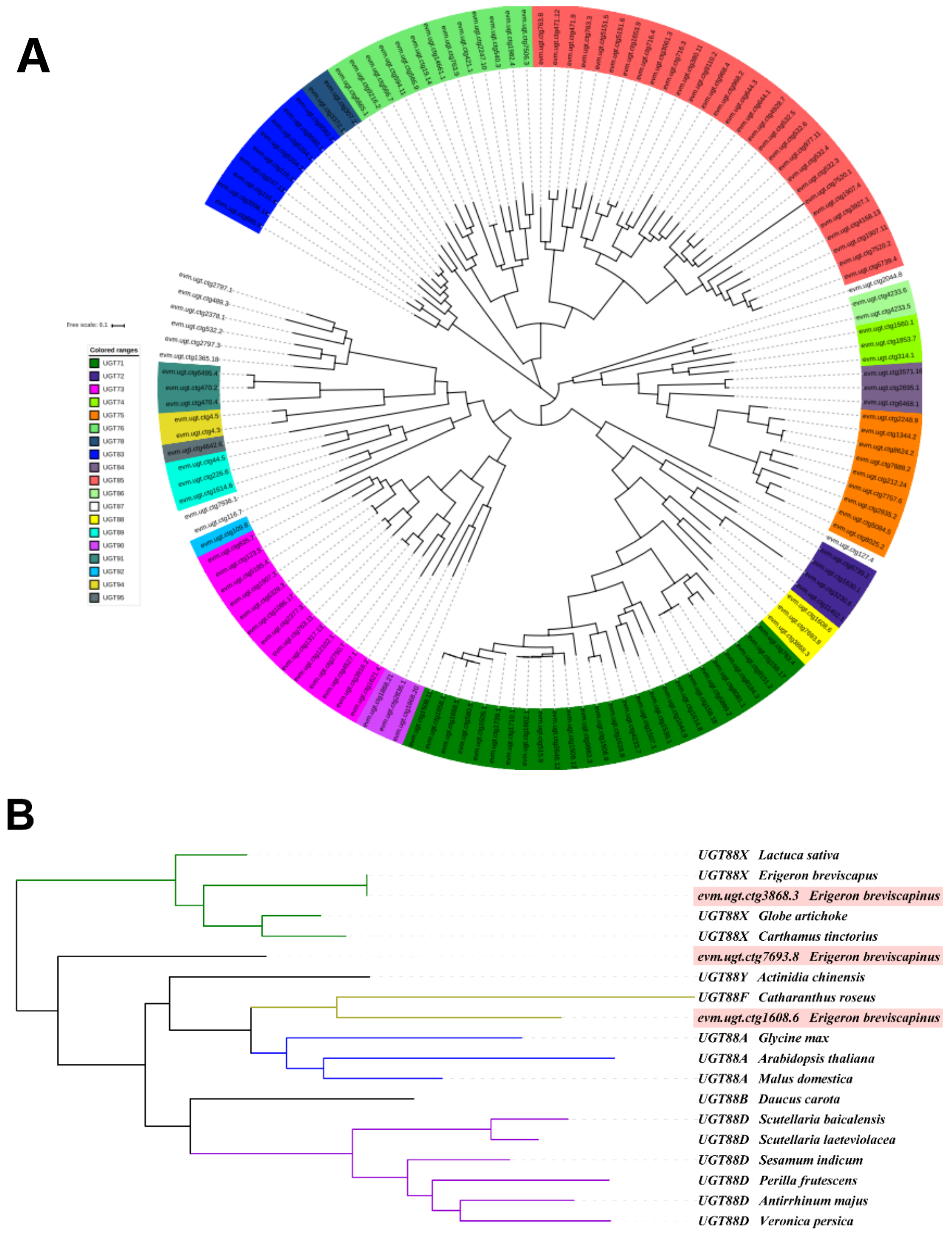

Figure S4. Phylogenetic tree of UDPGT. (A) The phylogenetic tree of 144 UDPGT genes from *E. breviscapus* genome. (B) The maximum-likelihood tree of UGT88 family.

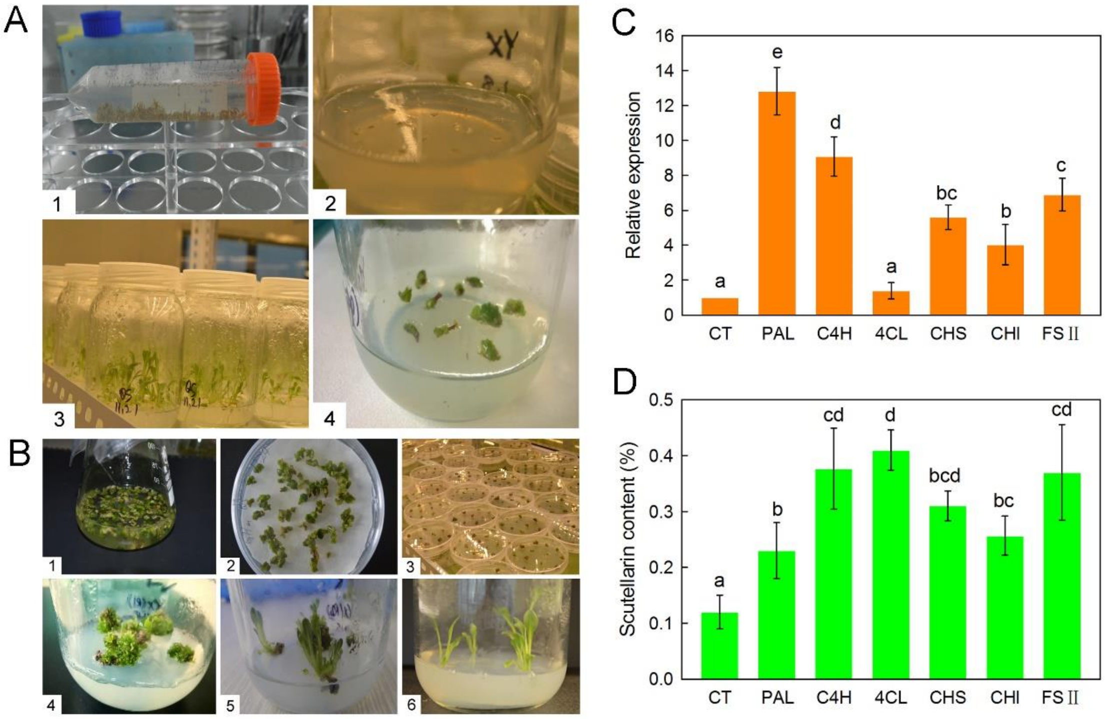

Figure S5. Transgenic *E. breviscapus* lines overexpressing PAL, C4H, 4CL, CHS, CHI, FSⅡ, respectively. (A) Establishment of the regeneration system. (1) Seed disinfection; (2) Inoculation; (3) Grow into plantlet; and (4) Callus was induced. (B) The transgenic process. (1 and 2) Inoculation and co-cultivation with Agrobacterium; (3) Selection of transgenic callus; (4 and 5) Differentiation; and (6) Regeneration. (C) Relative expression levels of six genes located upstream of breviscapine biosynthesis in transgenic *E. breviscapus* overexpression lines determined by qRT-PCR. (D) HPLC analysis of scutellarin content in overexpressing transgenic *E. breviscapus* lines.

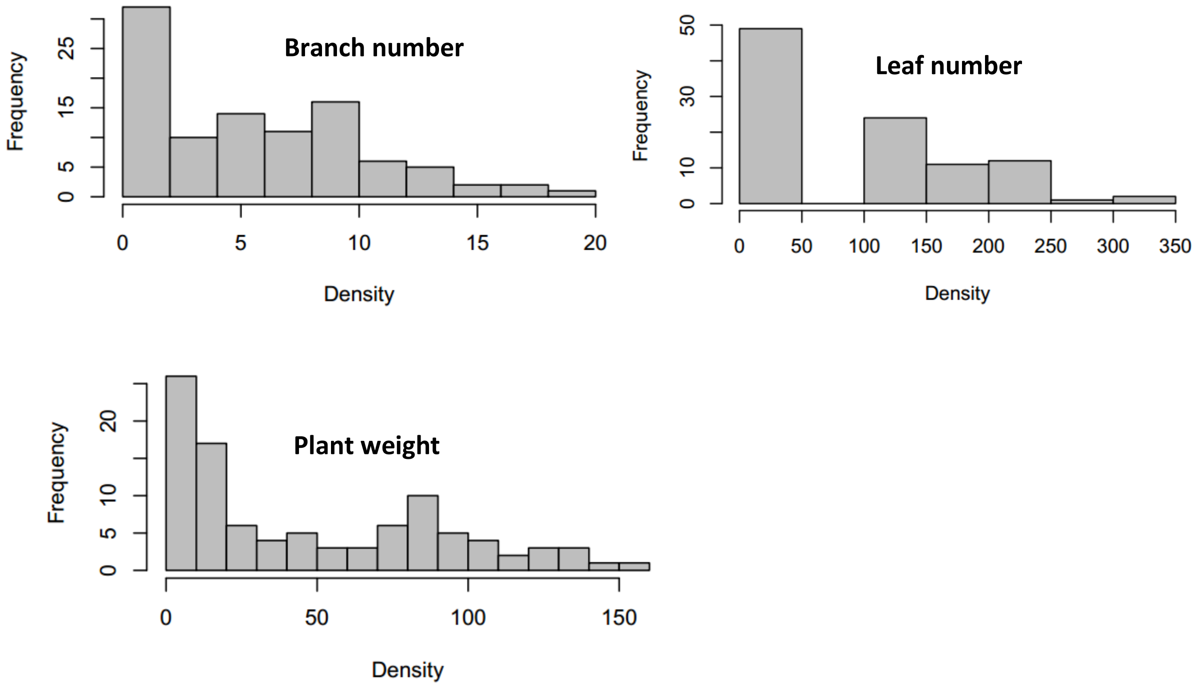

Figure S6. Frequency distribution of branch number, leaf number and plant height in *E. breviscapus.*

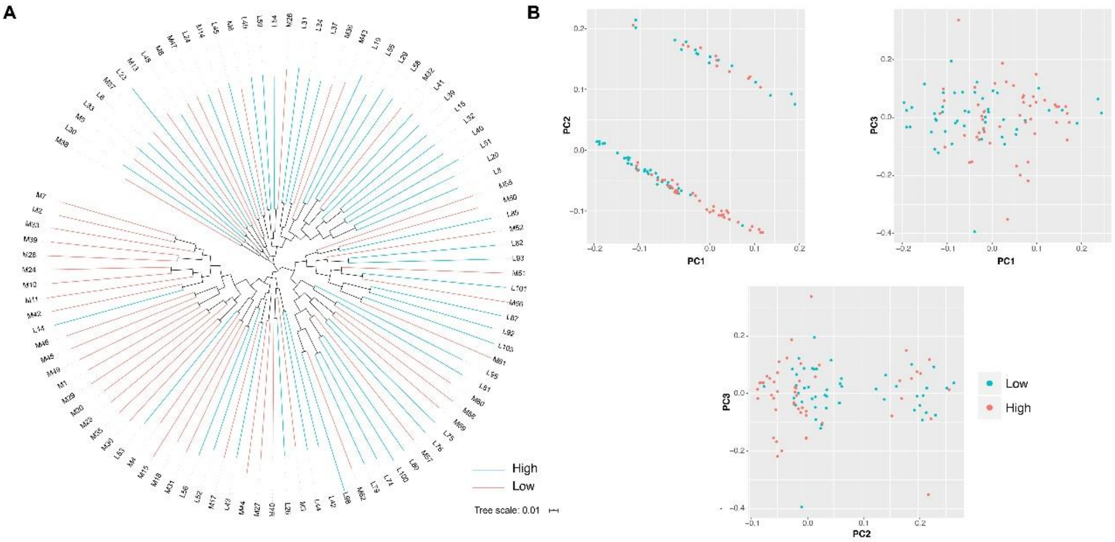

Figure S7. Population genetic analysis of 99 *E. breviscapus* individuals. (A) Phylogenetic tree and (B) principal component analysis. Low leaf number and high leaf number samples were colored in green and red, respectively.

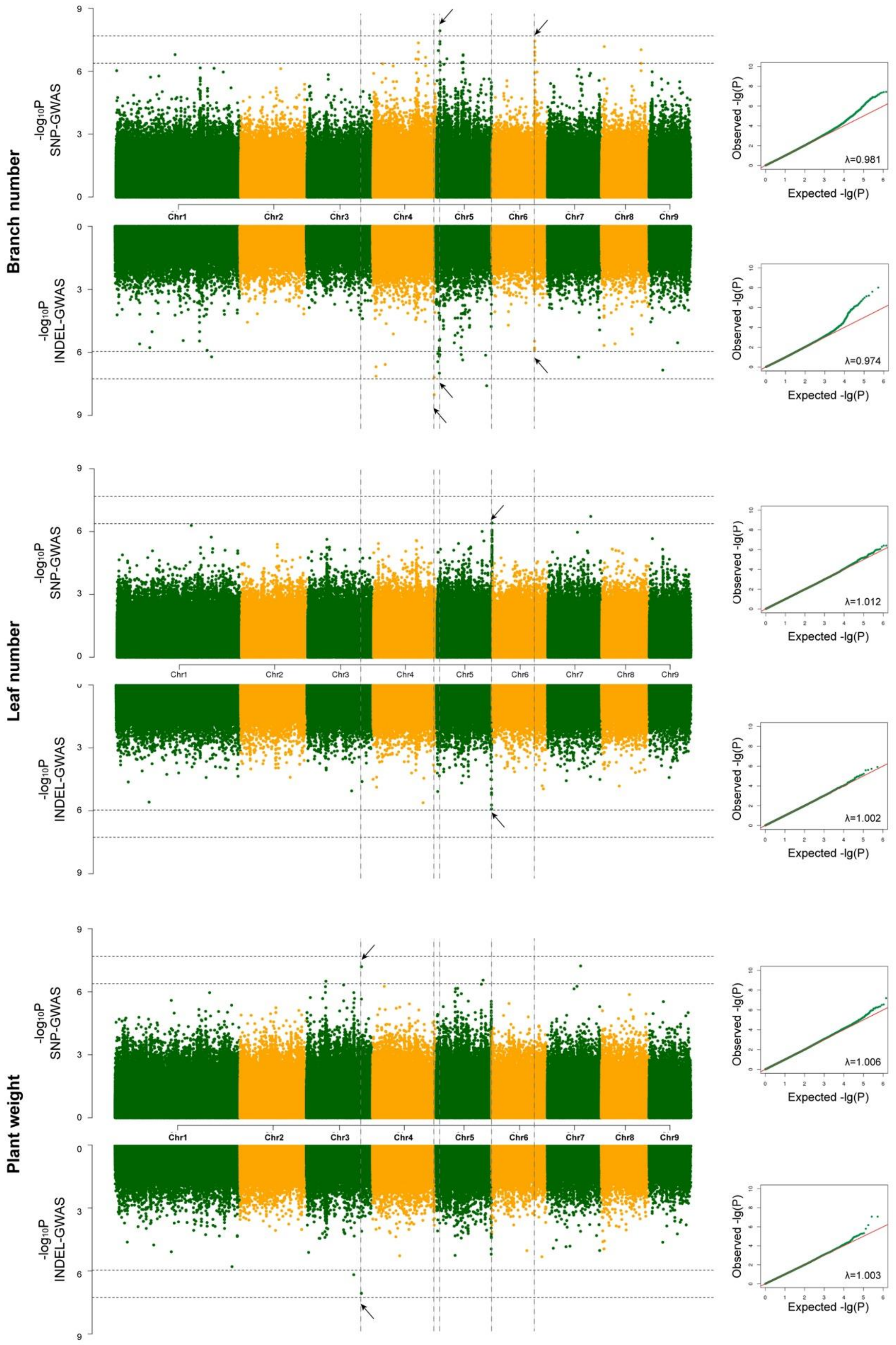

Figure S8. Manhattan and quantile-quantile plots for Genome-wide association studies of branch number, leaf number and plant height. For

each trait, SNP- and Indel-GWAS were plotted at the upper and lower part, respectively. Negative $\log_{10}$-transformed *P* values from the genome-wide scan were plotted against variants position on each of 9 chromosomes. Tow dotted horizontal line indicates the significant (0.05/N) and suggestive (1/N) P-value threshold, while N stands for the effective variant number. The genomic inflation factors (lambda) calculated by R pacage qqman were plot at the lower right corner of Q-Q plots. Five horizontal lines indicate strong peaks and arrows highlight strong signals.
